# How do brochures encourage walking in natural environments in the UK? A content analysis

**DOI:** 10.1093/heapro/daw083

**Published:** 2016-10-28

**Authors:** Lewis R Elliott, Mathew P White, Adrian H Taylor, Charles Abraham

**Affiliations:** 1 Psychology Applied to Health Group, University of Exeter Medical School, Exeter, UK; 2 European Centre for Environment and Human Health, University of Exeter Medical School, Exeter, UK; 3 Peninsula Schools of Medicine and Dentistry, Plymouth University, Plymouth, UK

**Keywords:** physical activity, nature, recreation, environment and public health, exercise

## Abstract

Although walking for leisure can support health, there has been little systematic attempt to consider how recreational walking is best promoted. In the UK, local authorities create promotional materials for walking networks, but little is known about whether they effectively encourage walking through persuasive messaging. Many of these materials pertain to walks in natural environments which evidence suggests are generally visited less frequently by physically inactive individuals. Consequently the present study explores whether and how recreational walking brochures use persuasive messages in their promotion of walks in natural environments. A coding taxonomy was developed to classify text in recreational walking brochures according to five behavioural content areas and 87 categories of potentially persuasive messages. Reliability of the taxonomy was ascertained and a quantitative content analysis was applied to 26 brochures collected from Devon, UK. Brochures often provided information about an advertised route, highlighted cultural and aesthetic points of interest, and provided directions. Brochures did not use many potentially effective messages. Text seldom prompted behaviour change or built confidence for walking. Social norm related information was rarely provided and there was a general lack of information on physical activity and its benefits for health and well-being. The limited range of message strategies used in recreational walking brochures may not optimally facilitate walking in natural environments for inactive people. Future research should examine the effects of theory-informed brochures on walking intentions and behaviour. The taxonomy could be adapted to suit different media and practices surrounding physical activity in natural environments.

## INTRODUCTION

Physical inactivity is increasing across Europe, threatening human health and costing the European economy over €80 billion per year (International Sport and Culture Association, 2015). Raising the physical activity levels of the less active members of the population is a public health priority, and promoting walking is potentially the most effective means of achieving this (Ogilvie *et al.*, 2007), in part because it is low cost and even normal walking pace can be health-enhancing (Rowe *et al.*, 2013). Importantly, natural environments appear to be locations which may be effective at encouraging health-enhancing bouts of walking. Survey research, for instance, suggests that individuals tend to spontaneously engage in longer episodes of physical activity, including walking, in natural rather than urban settings, and thus expend more energy on visits to these environments (Elliott *et al.*, 2015); while experimental research has demonstrated that people are more likely to conduct uninterrupted bouts of brisk walking in natural environments than in urban locations (Sellers *et al.*, 2012). Further, walking in natural environments can heighten affective benefits of walking compared to walking in urban settings (Thompson Coon *et al.*, 2011), leading to a greater likelihood that the activity will be repeated (Rhodes and Kates, 2015). Combined, these findings suggest that greater systematic efforts to promote walking in natural settings may play an important role in enhancing sustainable improvements in physical inactivity.

This is certainly the perspective of the UK’s National Institute for Health and Care Excellence (NICE, 2012), which identified the need for public health professionals to collaborate with colleagues in countryside management and park services in promoting walking among inactive individuals. Importantly, NICE also specified that there was a need to: “ensure programmes are based on an understanding of…factors influencing people's behaviour such as their attitudes, existing habits, what motivates them and their barriers to change” (NICE, 2012, p.14), and, “develop walking programmes for adults who are not active enough, based on an accepted theoretical framework for behaviour change” (NICE, 2012, p.18). In commenting on how these programmes should be promoted, NICE stated that programme directors should, “ensure programmes include communications strategies to publicize the available facilities (such as walking or cycling routes) and to motivate people to use them” (NICE, 2012, p.14).

The aim of the current research was to investigate the extent to which a sample of brochures promoting specific walks in natural environments in England, contain the kind of theoretically derived messages to motivate walking in natural settings that NICE recommends. In the UK, brochures advertising recreational walking (i.e. walking during free time for the purposes of enjoyment, Hurd and Anderson, 2011), in natural environments, are commonly produced by local authorities, councils, charities, and tourism organisations, and are aimed at both local residents and tourists/visitors (Hayes and MacLeod, 2007). Although we recognize that such walking leaflets may not have been produced as ‘health promotion materials’ *per se*, it is nonetheless informative to investigate whether they already contain many of the techniques suggested by theory, and whether such an investigation could provide insights into how future leaflets could be developed to include more theory-based techniques, in line with NICE’s recommendations, to motivate people to undertake more walks in the future.

An examination of walking brochures, in particular, makes sense because written materials are a widely used medium for communicating persuasive messages (Brito and Pratas, 2015), and promoting behaviour change (Bull *et al.*, 2001). They have also been found to be among the most effective tools in promoting walking programmes (Hunter *et al.*, 2015). Nevertheless, there is evidence that written materials advertising physical activity more generally are not always informed by behaviour change theory. For instance, one content analysis of 22 physical activity brochures identified a lack of messages relating to goal-setting, planning and affective benefits of physical activity (Gainforth *et al.*, 2011). The omission of such messages may mean these materials only motivate active people and may deter inactive people from taking up physical activity, which may be especially important in the case of walking, a relatively simple and cost-effective way to become less sedentary.

Nevertheless, to our knowledge, no content analysis of persuasive messages in recreational walking brochures has yet been undertaken. Consequently, our main task was to develop a relevant taxonomy of potentially persuasive message categories that could feasibly be contained within such brochures and then to identify their prevalence among a selected sample. To do this we adapted a pre-existing taxonomy developed for the analysis of health promotion materials. Our two main research questions were: a) Can the content of recreational walking brochures be reliably categorized?; and b) If so, what persuasive messages tend to be included in recreational walking brochures?

## METHODS

Specifically, we used the Content Analysis Approach to Theory-Specified Persuasive Educational Communication (CAATSPEC) (Abraham *et al.*, 2007) to inform the development of our coding taxonomy. CAATSPEC is an approach to quantitative content analysis of persuasive texts and can be used to outline messages used in health promotion materials. It uses mutually exclusive coding categories to classify content and was suited to this study as recreational walking brochures are (potentially) persuasive texts that promote a change in a health-related behaviour (the uptake of a specific walk). This is the first known application of CAATSPEC to materials in which health promotion may not necessarily have been the primary aim.

### Sampling

Brochures were collected from July to December 2013 in the county of Devon, UK. Convenience sampling was employed; sourcing brochures from councils, holiday parks, visitor information centres and supermarkets. This involved visiting as many of these places as was feasible in three principal holiday destinations (Exmouth, Dawlish and the north Devon coast) and one major city (Exeter). The following inclusion criteria were applied: a) the brochures existed in printed and digital form and advertized recreational walking in natural environments including mixtures of urban and natural environments and; b) brochures had to be available free of charge to ensure they could have the widest readership. While convenience sampling results in an unrepresentative sample, it is justified here as: (i) all possible printed recreational walking brochures in the county were difficult to obtain; (ii) it would have been extremely labour-intensive to have even attempted to do so, and; (iii) the current selection of brochures is still useful for generating hypotheses about the effectiveness of content in recreational walking brochures; three conditions necessary for selecting convenience sampling for quantitative content analysis (Riffe *et al.*, 2014, pp. 75-76). In total, twenty-six brochures were collected (see details in the Supplementary Material, S1). Brochures had a range of 54 to 712 paragraphs and 524 to 17,126 words (*M *= 3,539). They were associated with 29 different organisations and printed by nine different production companies. Two pages from a specific brochure are displayed in the supplementary materials for illustrative purposes (Supplementary Material, Figure S2).

### Taxonomy

**Fig. 1:**
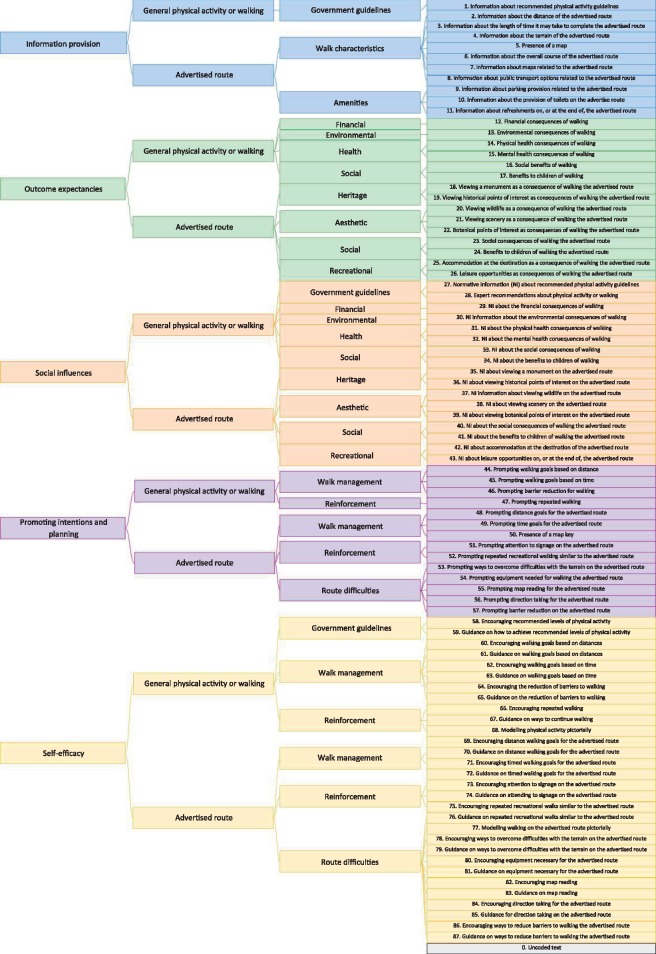
Hierarchical coding taxonomy.

Following initial readings, message categories corresponding to specific messages included in the brochures were devised. All categories were arranged under five superordinate headings which encompassed the key components of behaviour change in a variety of evidence-based theories, namely, *providing information, highlighting potential consequences and opportunities, establishing normative beliefs, promoting intentions and planning*, and *enhancing self-efficacy* (Albarracín *et al.*, 2005; Fisher and Fisher, 1992). In a previous application of CAATSPEC, the latter two superordinate headings were collapsed (Abraham *et al.*, 2007), but are separated here to highlight their exclusivity in conceptions of behaviour change (e.g. in the theory of planned behaviour). The final taxonomy had three further levels of specificity arranged hierarchically and can be viewed in Figure 1.

We attempted to categorize brochure text into message categories using established taxonomies of behaviour change techniques (Abraham and Michie, 2008; Michie *et al.*, 2013). A taxonomy emerged where each category represented a distinct potentially persuasive message. However, categories warranted greater specificity than techniques defined in general taxonomies. To take an example, Abraham and Michie identified the general change technique “provide information on consequences” as derived from explanatory theories (Abraham and Michie, 2008). The authors defined the technique as, “information about the benefits and costs of action or inaction, focusing on what will happen if the person does/does not perform the behaviour.” (p.382). This technique was rendered domain-specific by Michie and colleagues (Michie *et al.*, 2013) who identified the technique as comprising health, social, environmental, and emotional consequences (p.92). In the present study, we further adapted the technique to better represent persuasive messages found in recreational walking brochures. Specifically, consequences of recreational walking in the present taxonomy comprised health, social, environmental, financial, heritage, aesthetic, and recreational consequences (see definitions below).

In a similar way to previous applications of CAATSPEC (Gainforth *et al.*, 2011), categories were created to classify pictures of people walking (modelling behaviour) and graphics of maps (aids to planning). Listed below are details of categories under each superordinate from the finalized taxonomy. The full coding manual can be viewed in the supplementary materials (Supplementary Material, S3).

### Providing information

Category 1 reflected information on PA recommendations or the prevalence of PA or walking in a population. Categories 2-7 detailed characteristics of the route such as the terrain or distance. Categories 8-11 concerned amenities such as public transport or refreshments on the route.

### Highlighting potential consequences and opportunities

Categories 12-17 concerned general consequences of PA or walking including: financial (e.g. saving money over car trips); environmental (e.g. sustainable travel mode); physical and mental health (e.g. improving cardiovascular health; feeling happier); and social (e.g. family enjoyment). Categories 18-26 described opportunities on the advertised route such as heritage (e.g. historical sites); aesthetics (e.g. wildlife, scenery); social (e.g. opportunities for children’s enjoyment); and recreation (e.g. leisure opportunities).

### Establishing normative beliefs

Categories 27–34 outlined normative information about PA or walking, or the consequences of these including: expert recommendations on PA, and financial, environmental, health, and social consequences. In a similar way to highlighting potential consequences and opportunities, categories 35–43 detailed normative information about opportunities related to walking the advertised route.

### Promoting intentions and planning

Categories 44–47 prompted behaviours related to PA or walking including: setting goals based on distances (e.g. decide how far you will walk); or times (e.g. consider freeing up some time for walking); reducing barriers (e.g. think what would make being active easier for you); or prompting activity maintenance (e.g. make sure to ‘keep up’ your walking once you have started). Categories 48-57 identified messages specific to the advertised route such as prompting goals based on distance (e.g. try breaking up the route into segments); attending to signage (e.g. use the waymarkers); or managing the terrain (e.g. be careful of the busy road).

### Enhancing self-efficacy

Following CAATSPEC, most categories under this superordinate were dichotomized as *encouraging* or *guiding* behaviour. *Encouragement* categories conveyed that behaviour was easy to execute, and *guidance* categories instructed on how to execute behaviour. Categories 58–68 related to building confidence for PA or walking in general and included: guidance on reducing barriers to activity, for example not knowing where to walk (e.g. go to a website and you can find guided walks in your area); encouraging setting walking goals based on time (e.g. it is easy to find everyday opportunities to go walking); or modelling walking pictorially. Categories 69–87 related to building confidence for completing the advertised route and included: guidance on maintaining recreational walking behaviours (e.g. purchase more outdoor walking brochures from the visitor information kiosk in the city centre); encouraging the use of appropriate equipment (e.g. it is simple to get walking boots from your local outdoors shop); or guidance on direction taking (e.g. turn left at the end of the road). As can be imagined, this last category was likely to be central to recreational walking brochures.

### Coding procedures

A pilot coding manual was tested by two coders but demonstrated insufficient reliability. To improve the manual, categories were added and deleted, definitions were revised, and coding procedures were modified. With the revised manual, and in accordance with previous research (Gainforth *et al.*, 2011), a line-by-line coding procedure was utilized to facilitate inter-coder reliability testing. Sentences acted as ‘units of analysis’ and coders were instructed on how to detect semantic changes within and across sentences, and how to code these. Categories were exclusive; text could only be coded under one category. The manual also provided guidance on distinguishing semantically similar categories. For example, some messages *prompted* behaviours whilst others provided *guidance* on the same behaviours e.g. category 53 refers to messages suggesting ways to deal with the terrain on the advertised route whereas category 79 refers to messages explicitly providing guidance on how to deal with these. Coders were instructed that any category *prompting* behaviour will refer to specific behaviour (e.g. be careful climbing the muddy hill) but any category *guiding* behaviour will inform them on *how to* execute that behaviour (e.g. taking shorter strides will ensure you do not slip up on the muddy hill). Coding instructions can be seen in the supplementary materials (Supplementary Material, Figure S3). Coding a brochure took approximately 90 minutes.

### Reliability

Inter-coder reliability was assessed using the *AC1* statistic (Gwet, 2002). The prevalence of some categories was very small and *AC1* adjusts reliability accordingly where alternatives (e.g. Cohen’s Kappa) (Cohen, 1960) would not. The protocol for reliability testing was as follows: Two brochures were selected by the first author on the basis that they varied in style, length and publisher; thus potentially encompassing the broadest range of categories. Two coders (including the first author) would code the brochures, line-by-line, as described above. If reliability was established at all hierarchical levels (*AC1 *≥* *0.7, *p* < 0.05), testing would stop, providing that individual categories demonstrated reasonable reliability too (*AC1 *≥* *0.6; *p* < 0.2). This generous alpha level was selected so that categories with only one agreed instance (identified by both coders) were judged reliable despite the lack of more instances to determine reliability at conventional alpha levels. This is because coders selecting one piece of text and identifying it as the same category of a possible 87 was unlikely to be due to chance. If any individual categories did not meet this criterion, consensus would be sought using an independent coder (the second author) and the category removed if agreements on disagreed instances were not reached. If any level of the hierarchy demonstrated unsatisfactory reliability, the manual would be revised and testing repeated with two further brochures. If any individual category’s *AC1* exceeded the alpha level (*p *>* *0.2), or if there were no instances of a category found, the category was deemed a ‘potential category of persuasive message’, but with insufficient data to determine reliability.

### Analysis strategy

To examine frequently employed persuasive messages, only categories which appeared in more than three brochures were included in the main analysis. Categories which appeared in more than three brochures but had insufficient data to determine reliability in the testing phase were noted as requiring further reliability testing. We examined frequencies and proportions of content firstly across and then within superordinate categories.

## RESULTS

### Reliability

Consult supplementary materials (Supplementary Material, S4) for reliability statistics. 476 category instances (9.3% of all content) were double-coded. Coders agreed on the same categories for 363 (76.26%) of these. Satisfactory reliability was achieved at all levels of the hierarchy (superordinate level: *AC1 *=* *0.77, 95% CI 0.73, 0.82; individual category level: *AC1 *=* *0.76, 95% CI 0.72, 0.80). There were only 35 categories (including an ‘uncoded text’ category) that contained enough instances to confirm reliability with a statistically significant *AC1* value. We believe this reflects the lack of diverse persuasive messages used in brochures and not inadequate sampling. The number of additional categories for which reliability could have been established through double-coding more brochures did not justify the labour involved in further line-by-line double-coding.

There were six categories that did not meet our reliability criteria (*AC1 *≤* *0.6; *p *<* *0.2). All instances coded under these categories were discussed between the first and second author, and categorisations agreed for all, so no categories were removed. Afterwards, 448 of the 476 category instances were agreed upon and the reliability of all levels of the hierarchy had improved significantly (superordinate level: *AC1 *=* *0.96, 95% CI 0.94, 0.98; individual category level: *AC1 *=* *0.94, 95% CI 0.92, 0.96). As a consequence of this resolution phase, two further categories did not meet our reliability criteria (category 53: *prompting ways to overcome difficulties with the terrain on the advertised route*; category 73: *encouraging attention to signage on the advertised route*). In total these categories only comprised five disagreements, so in line with previous content analyses (Abraham *et al.*, 2007), decisions of the first author were accepted as they had the benefit of coding all brochures in the sample.

### Content analysis

All percentages reported reflect subordinate categories which were included in more than three brochures in the sample. Using this criterion, 33 of the original 87 categories formed a useful taxonomy of potentially persuasive messages frequently used in recreational walking brochures. Descriptive statistics for these 33 categories are displayed in Table 1; the supplementary materials (Supplementary Material, S5) contain descriptive statistics for all categories. Of these 33, seven had insufficient data in reliability phase to determine reliability (categories 3, 18, 49, 55, 70, 77, and 81) and another was category 53, which, as discussed earlier, did not meet the 0.6 *AC1* threshold after the resolution phase. Interpretations on all of these categories should therefore be considered cautiously. Of the 25 with sufficient data in the reliability phase, *AC1*’s ranged from 0.69 to 1.00, so good reliability can be assumed for the rest of the categories included here. There were 4,800 instances of coded text within these 33 categories (94% of all content). Messages providing information accounted for 30.92% of all coded content (*M *=* *57 instances per brochure). Messages highlighting consequences accounted for 26.94% (*M *=* *50 instances). Messages promoting intentions and planning accounted for 5.58% (*M *=* *10 instances). Messages enhancing self-efficacy accounted for 36.56% (*M *=* *68 instances). No categories pertaining to messages establishing normative beliefs appeared in more than 3 brochures.

**Table 1:** Frequency of category inclusion

Message	No. of brochures	No. of instances	Max instances	% all content	% of superordinate
Providing information	26	1484	299	30.92	–
2. Information on the distance of the advertised route	24	221	44	4.60	14.89
3. Information on the length it may take to complete the advertised route	7	27	14	0.56	1.82
4. Information on the terrain of the advertised route	22	228	48	4.75	15.36
5. Presence of a map	22	106	12	2.21	7.14
6. Information on the overall course of the advertised route	23	393	91	8.19	26.48
7. Information on maps related to the advertised route	13	54	19	1.13	3.64
8. Information on public transport options related to the advertised route	16	254	54	5.29	17.12
9. Information on parking provision related to the advertised route	10	42	9	0.88	2.83
10. Information about provision of toilets on the advertised route	12	47	18	0.98	3.17
11. Information on refreshments on, or at the end of, the advertised route	15	112	29	2.33	7.55
Highlighting consequences	26	1293	361	26.94	–
18. Viewing a monument as a consequence of walking the advertised route	13	44	13	0.92	3.40
19. Viewing historical features as consequences of walking the advertised route	26	663	187	13.81	51.28
20. Viewing wildlife as a consequence of walking the advertised route	20	85	20	1.77	6.57
21. Viewing scenery as a consequence of walking the advertised route	24	300	98	6.25	23.20
22. Botanical points of interest as consequences of walking the advertised route	20	60	13	1.25	4.64
25. Accommodation at the destination as a consequence of walking the advertised route	6	17	6	0.35	1.31
26. Leisure opportunities as consequences of walking the advertised route	11	124	30	2.58	9.59
Promoting intentions and planning	26	268	55	5.58	–
48. Prompting distance goals for the advertised route	7	16	5	0.33	5.97
49. Prompting time goals for the advertised route	6	13	6	0.27	4.85
50. Map key	17	25	2	0.52	9.33
51. Prompting attention to signage on the advertised route	10	27	8	0.56	10.07
52. Prompting repeated recreational walking similar to the advertised route	19	105	17	2.19	39.18
53. Prompting ways to overcome difficulties with the terrain on the advertised route	9	37	12	0.77	13.81
55. Prompting map reading for the advertised route	5	7	3	0.15	2.61
56. Prompting direction taking for the advertised route	5	7	2	0.15	2.61
57. Prompting barrier reduction on the advertised route	9	31	13	0.65	11.57
Enhancing self-efficacy	26	1755	403	36.56	–
70. Guidance on distance walking goals for the advertised route	7	11	3	0.23	0.63
74. Guidance on attending to signage on the advertised route	5	5	1	0.10	0.28
76. Guidance on repeated recreational walks similar to the advertised route	12	65	20	1.35	3.70
77. Modelling walking on the advertised route pictorially	8	35	12	0.73	1.99
79. Guidance on ways to overcome difficulties with the terrain on the advertised route	14	50	8	1.04	2.85
81. Guidance on equipment necessary for the advertised route	4	6	3	0.13	0.34
85. Guidance for direction taking on the advertised walk	23	11	362	32.98	90.20

“No. of brochures” refers to the number of brochures in which the category (or superordinate content area) featured.

“No. of instances” refers to the number of instances of the category (or superordinate content area) that were present in all 26 brochures.

“Max instances” refers to the maximum number of instances of the category (or superordinate content area) in any one brochure.

“% all content” refers to the percentage of all content (encompassed by these 33 categories) which is accounted for by the category (or superordinate content area).

“% of superordinate” refers to the percentage of superordinate content which is accounted for by the category.

### Messages providing information

The most prevalent messages under this superordinate were those categorized as *information about the overall course of the advertised route* (category 6), accounting for 26.48% of all content which provided information, and 8.19% of content overall. This included summaries of where the route would take the reader e.g. ‘this walk explores an inland section of the Bude Canal on the Devon-Cornwall border’ or information on the location e.g. ‘Exmouth is a gateway town’. Other widely used categories included *information about public transport options related to the advertised route* (category 8) e.g. ‘many of the trails have convenient parallel public transport routes - bus or train’, *information about the terrain of the advertised route* (category 4) e.g. ‘mostly level and easy although there is one steep climb on an inclined plane’, and *information about the distance of the advertised route* (category 2) e.g. ‘a 13km/8 mile circuit’.

### Messages highlighting potential consequences and opportunities

The most frequently occurring types of messages were those categorized as *viewing historical points of interest as consequences of walking the advertised route* (category 19) accounting for 51.28% of content which highlighted consequences and 13.81% of content overall. This was also the only category to appear in every brochure. This incorporated descriptions of geology e.g. ‘celebrating 95 miles of internationally important rocks displaying 185 million years of the Earth's history, the Jurassic Coast is a geological walk through time’. It also detailed historical facts about the advertised route e.g. ‘in 1861, the arrival of the railway, linking the town with Exeter, brought with it a dramatic population explosion’. Other common categories included *viewing scenery as a consequence of walking the advertised route* (category 21) e.g. ‘the South West Coast Path is a superb way to experience a range of fine Devon scenery, from cliff tops to wide estuaries, sandy bays to wooded valleys’, and *leisure opportunities as consequences of walking the advertised route* (category 26) e.g. ‘the estuary is a hub of activity for recreational activities; such as sailing, canoeing, windsurfing, fishing and scuba diving’.

### Messages promoting intentions and planning

*Prompting repeated recreational walking similar to the advertised route* (category 52) was the most utilized message category, responsible for 39.18% of promoting intentions and planning content and 2.19% of content overall. This included the promotion of related brochures *without* instruction on how to obtain these e.g. ‘an introductory leaflet and a detailed route book on the Tarka Trail are both available’. It also included ways to enjoy the advertised walk, again *without* instruction on how to do so e.g. ‘why not try your hand at Geocaching when on the trail’? It further included contact details on guided walks e.g. ‘why not join one of a number of free guided tours’? Another often used category was *prompting ways to overcome difficulties with the terrain on the advertised route* (category 53). This included directions to ‘be aware’ or ‘take care’ e.g. ‘care should be taken at all times when walking on roads’, or, ‘take care crossing the Exe river over Bickleigh Bridge’. Another common category was *prompting barrier reduction on the advertised route* (category 57) e.g. ‘you can pick up short sections of the trail from a number of easily accessible points’.

### Messages enhancing self-efficacy

The most often used category was *guidance for direction taking on the advertised walk* (category 85). This category was present in 23 of the brochures and accounted for 90.20% of all self-efficacy content, and 32.98% of content overall. It embodies the nature of walking brochures; instructing on how to progress through a route. This is different from the provision of route information as it builds confidence for wayfinding. Examples include, ‘just before you reach a cattle grid turn left alongside a bank’, or, ‘go through the gate at the top left corner of the next field, to the road’. In a similar way to messages promoting intentions and planning, other common categories included *guidance on repeated recreational walks similar to the advertised route* (category 76). This is different from the promotion of repeated recreational walks as it provides means by which the reader can access further walking information. For example, ‘free booklets about Devon’s coast and countryside including walking trails, cycling, horse riding and wildlife can be ordered through the Devon County Council website at www.devon.gov.uk’, or, ‘leaﬂets on all of these walks are available from Exeter City Council and the Visitor Information Centre’. Other frequently used message categories were *guidance on ways to overcome difficulties with the terrain on the advertised route* (category 79) e.g. ‘this route is closed during the shooting season from 1st October to 1st February, and walkers should follow the alternative route along the quiet road instead at that time’, or, ‘aim to walk this part of the route within two hours of low tide (see local press or visit www.teignestuary.org)’, and *modelling walking on the advertised route pictorially* (category 77).

### Uncategorized content

4.04% of all content was unable to be categorized under any of the 87 categories. This equated to 206 instances of uncategorized text compared to 4,893 instances of categorized text. The proportion of text which went uncategorized per brochure ranged from 0% to 10.71%. Examination of this text revealed no systematic exclusion of content related to recreational walking. The majority of this text related to authorship credits, website addresses unrelated to walking, and advertisements for holiday attractions. The only recurring behavioural message types that went uncategorized concerned the advertisement of cycle routes and the prompting or instructing of environmental behaviours e.g. ‘support local shops and services’ or, ‘take your litter home and recycle it where possible’.

## DISCUSSION

This is the first known study to develop a specific coding taxonomy for, and conduct a content analysis of, recreational walking brochures. Acceptable reliability of this taxonomy was established at each hierarchical level and for most frequently occurring categories. The content analysis suggested that brochures promoted walking in natural environments through messages which provided information on the route, highlighted potential consequences and guided on wayfinding. However, they lacked variety in message types; frequently omitting information which could raise normative beliefs, promote intentions, or enhance self-efficacy, for walking.

### How do brochures encourage recreational walking in natural environments?

Brochures often provided information that aimed to facilitate easier access to a walking route, as opposed to information about PA more generally. They also provided information on the course, distance, duration, and terrain of a route, seemingly in order to detail the amount of time and level of expertise required to undertake the walk. In contrast to traditional PA promotion, messages highlighting consequences often framed scenic features as reasons to walk rather than potential health gains. Importantly, previous research has demonstrated that for people who visit natural environments infrequently, subjective qualities like this are more important motivators for visiting than the achievement of physical fitness (Dallimer *et al.*, 2014). Thus, highlighting these may persuade less frequent visitors, who are also more likely to be less active (Coombes *et al.*, 2010), to visit natural environments. Promoting intentions and enhancing self-efficacy in the brochures mainly drew the reader’s attention to other recreational walking materials and how to access them. This could support walking maintenance behaviours, but the aim of those messaging strategies may have been simply to drive further interest in a destination or organisation.

### Do brochures conform to NICE guidance on walking promotion?

A public health priority is to encourage those who are least motivated, to engage in recreational walking (Ogilvie *et al.*, 2007), and natural environments could support this. Considerable investment has been directed towards improving environments and opening walking routes (Hunter *et al.*, 2015) but little is known about how to sell these opportunities through printed media to those who are less motivated to walk. In the present study, walking brochures lacked general and normative information about PA for health, behavioural prompts and efficacy information (especially content encouraging general walking behaviours). Messages containing such information can be effective in motivating inactive people to set better plans to undertake PA (Sweet *et al.*, 2014). Most brochures and much of the content therein, whether intentionally or not, was therefore intended for people who already do recreational walking in the natural environment. This is at odds with guidance on walking promotion (NICE, 2012). While further research is needed to explore which messages are most effective, there appears to be more scope in the brochures to change cognitions about recreational walking (e.g. build confidence to complete walks, raise descriptive norms about outdoor walking), and encourage behavioural strategies (e.g. provide walking goals in terms of distance or time). Doing so would help meet NICE’s recommendation that local authorities “develop walking programmes for adults who are not active enough, based on an accepted theoretical framework for behaviour change” (NICE, 2012, p.18).

An example of how to achieve this is illustrated in one of the brochures in the sample. Exeter Walking Map stood out as the brochure having both the highest category-to-instance ratio (24 categories featured comprising 51 textual instances) and the most even distribution of categories across superordinate content areas. This brochure was also devoted to the promotion of walking more generally as opposed to its related recreational walking routes (around the city of Exeter, UK). For example, it outlined physical health consequences (category 14) e.g. ‘walking can help you live longer, helps protect you from heart disease, diabetes, cancer, osteoporosis and much more’ and included four references to mental health consequences (category 15) e.g. ‘walking can activate the happy hormone which makes you feel good, improves your mood and reduces stress’. It contained normative information on benefits to children (category 34) e.g. ‘children like to walk to school so they can chat to their friends.’ Furthermore, it included text reducing general barriers to walking (category 46) e.g. ‘walking need not require any special equipment’, and provided guidance on walking goals based on time management (category 63) e.g. ‘by walking to work, school, the shops or the station you can get your daily exercise as part of your normal routine’. It was also one of only two brochures in the sample to state PA guidelines; in this case providing guidance on how someone could achieve them (category 59): ‘Doing 10,000 steps per day will contribute to the recommendation of moderate-intensity physical activity for at least 30 minutes on 5 or more days per week’. This brochure demonstrates how a variety of theory-derived persuasive messages can be incorporated into a recreational walking brochure. Naturally, many more considerations are involved in creating a brochure. The overall layout, typesetting, language style and numerous other features are important in attracting or deterring a potential reader from picking up a brochure or persuading them to change their behaviour (Abraham and Kools, 2012). Nonetheless, the selection of appropriate behavioural antecedents to write into messages remains important (Brawley and Latimer, 2007).

### Strengths, limitations, and future research

The main strength of this study is that it produced a flexible taxonomy for analysing materials that advertise recreational PA in a variety of different communication channels such as websites or mobile applications. Furthermore, it has identified for the first time the range of messages used in walking brochures which attempt to attract people to recreate in certain landscapes. The coding taxonomy was designed to facilitate easier analysis of other recreational PA materials by maintaining stable superordinate content areas within which users could define individual categories to suit different environments, PA conventions and cultures. Notwithstanding their geographical specificity, the sample of brochures did nonetheless cover a variety of environments (coastal, rural, city) near smaller and larger conurbations.

Some of the brochures detailed long-distance trails. While long-distance trails traverse many settlements, they tend not to locate near to larger conurbations meaning that they may not facilitate everyday recreational walking for populations such as those living in urban areas of high deprivation who experience a greater burden of inactivity-related poor health (Ball, 2015). Focusing on how to best promote shorter-distance recreational walking in urban green spaces may be more effective in ameliorating the relative lack of greenspace use by these populations (Jones *et al.*, 2009). While convenience sampling was employed to generate hypotheses about the effectiveness of brochure content, if feasible, future content analyses of recreational walking materials may wish to employ probability sampling methods to ensure better representativeness.

Although the taxonomy was reliable at all levels of the hierarchy, reliability for eight frequently occurring categories could not be established. While this suggests inadequate sampling, not one of these categories alone accounted for more than 1% of all content, suggesting that further reliability testing may still not have yielded enough instances for confident reliability assessments. Perhaps in the future a combination of traditional presence-or-absence methods (e.g. Abraham *et al.*, 2007) supplemented by line-by-line procedures (e.g. Gainforth *et al.*, 2011) could improve reliability protocols in comparable content analyses. Nevertheless, categories may need to be omitted or revised in any future applications of the taxonomy should they fail to meet acceptable reliability criteria.

Developing the categories in the present taxonomy was achieved in part by expanding behaviour change techniques from other taxonomies (Abraham and Michie, 2008; Michie *et al.*, 2013). This suggests that in any context-specific content analysis, especially those examining materials where health promotion is perhaps a secondary aim, such taxonomies could possibly only be used to derive more relevant message categories. Even with the present taxonomy, categories such as *mental health consequences of walking* (category 15) could be subdivided into affective benefits, restorative benefits, and spiritual benefits for instance. Each may be differently persuasive for different readers. In the future, researchers must consider the strengths of comprehensiveness and parsimony when deciding upon message categories.

In future, controlled trials could use the taxonomy prospectively as a guide to creating intervention materials that target different antecedents of behaviour change, and test with more precision which ‘ingredients’ are most effective and appealing to different groups (eg, urban *vs.* rural dwellers, tourists *vs.* home based, disadvantaged *vs.* affluent communities). Future research might also wish to test different types of brochure in terms of their ability to alter attitudes towards walking or intentions to walk. For example, controlled studies could administer brochures which were identical in style but varied in terms of the type of message employed. This would allow researchers to test how original *vs.* tailored information could be differently persuasive and thus inform guidelines on how to produce recreational walking brochures.

## CONCLUSION

Content in recreational walking brochures sampled from Devon, UK, was coded for the presence of potentially persuasive messages using the coding taxonomy developed here. These brochures’ principle persuasive strategies are to guide wayfinding, provide information on amenities and access, and enhance the appeal of various properties of natural environments. Whilst highlighting attractive properties could motivate inactive people, omitting messages related to the promotion of intentions or self-efficacy and failing to raise normative beliefs may fail to encourage inactive people to engage in recreational walking in natural environments. In future, brochures could utilize a wider variety of message strategies in their text in order to engage such populations. Public health bodies could support the creation of recreational walking brochures to achieve this.

## SUPPLEMENTARY MATERIAL

Supplementary material is available at *Health Promotion International* online.

## FUNDING 

This work was supported by an Economic and Social Research Council (ESRC) doctoral studentship [Award Number: ES/J50015X/1] as part of the South-West Doctoral Training Centre (SWDTC) strategic partnership. It was also partly funded by the NIHR Leadership in Applied Health Research and Care of the South West Peninsula (PenCLAHRC). The European Centre for Environment and Human Health is financially supported by the European Regional Development Fund (ERDF) and the European Social Fund (ESF) Convergence Programme for Cornwall and the Isles of Scilly. The views expressed in this paper are those of the authors and not necessarily those of the ESRC, SWDTC, NIHR, UK Department of Health, ERDF or ESF. None of these were involved in data analysis or interpretation and bear no responsibility for the analyses or interpretations presented here.

## Supplementary Material

Supplementary File S-1

Supplementary File S-2

Supplementary File S-3

Supplementary File S-4

Supplementary File S-5
