# Supplementary material for: How do brochures encourage walking in natural environments in the UK? A content analysis
Source: Health Promot Int. 2016 Oct 28;33(2):299–310. doi: 10.1093/heapro/daw083 (PMC5892139; doi:10.1093/heapro/daw083)
Supplement: Supplementary File S-1 [file daw083_supplementary_file_s-1.docx]

| **Title** | **Publishing Affiliations** | **Producer** | **Pages** | **No. of walking routes** |
| --- | --- | --- | --- | --- |
| Avocet Ambles | Avocet Line Rail Users Group  Devon County Council  First Great Western | Avocet Line Rail Users Group | 4 | 3 |
| Bude Canal Trails | Discover Devon  North Cornwall District Council  CrossCut (Sustainable Development of Inland Waterways) | Devon Design & Print | 2 | 1 |
| Dart Ferries Walk, Pines Pebbles and Plantations & Torridge Estuary Rail Trail | Devon County Council  Travelwise | Devon Design & Print | 6 | 3 |
| Devon Cliffs to Budleigh | Devon Cliffs Holiday Park  South West Coast Path Association | South West Coast Path Team | 2 | 1 |
| Devon Cliffs to Exmouth | Devon Cliffs Holiday Park  South West Coast Path Association | South West Coast Path Team | 2 | 1 |
| Devon Cliffs to Orcombe Point | Devon Cliffs Holiday Park  South West Coast Path Association | South West Coast Path Team | 2 | 1 |
| Devon Cliffs to Otterton Mill | Devon Cliffs Holiday Park  South West Coast Path Association | South West Coast Path Team | 2 | 1 |
| Discovery Trail | Tamar Valley AONB  Devon County Council  Cornwall Council  Natural England | *Not stated* | 12 | 5 |
| Drake's Trail | Devon County Council  West Devon Borough Council  Plymouth City Council  Sustrans  National Trust  Dartmoor National Park | Graphic Words | 3 | 1 |
| Exe Explorer | Exe Estuary Management Partnership  Devon County Council  Exmouth Town Council  Dawlish Town Council  East Devon District Council  Teignbridge District Council  Natural England  Royal Society for the Protection of Birds | Exe Estuary Management Partnership | 2 | 1 |
| Exeter Green Circle - The Alphin Brook Walk | Exeter City Council  Devon County Council  Sustrans | Daniel Loveday | 2 | 1 |
| Exeter Green Circle - The Hoopern Valley Walk | Exeter City Council  Devon County Council  Sustrans | Daniel Loveday | 2 | 1 |
| Exeter Green Circle - The Ludwell Valley Walk | Exeter City Council  Devon County Council  Sustrans | Daniel Loveday | 2 | 1 |
| Exeter Green Circle - The Mincinglake Walk | Exeter City Council  Devon County Council  Sustrans | Daniel Loveday | 2 | 1 |
| Exeter Green Circle - The Redhills Walk | Exeter City Council  Devon County Council  Sustrans | Daniel Loveday | 2 | 1 |
| Exeter Medieval Trail | Exeter City Council | Meridian Print & Marketing | 2 | 1 |
| Exeter Walking Map | Exeter City Council  Devon County Council  Sustrans  Travelwise | Cycle City Guides | 2 | 1 |
| Exeter Woollen Trail | Exeter City Council | Meridian Print & Marketing | 2 | 1 |
| Explore Exmouth | Devon County Council  Sustrans  Travelwise | Devon Design & Print | 32 | 8 |
| Tarka Trail Circular Routes | Devon County Council  Travelwise | Corporate Communications: Design Service | 28 | 11 |
| The City Wall Trail | Exeter City Council | Meridian Print & Marketing | 2 | 1 |
| The Exe Valley Way | Discover Devon  Devon County Council  Exmoor National Park  Travelwise | *Not stated* | 28 | 10 |
| Two Castles Trail Booklet | Devon County Council  West Devon Borough Council  Dartmoor National Park | Graphic Words | 24 | 8 |
| Two Moors Way | Devon County Council  South Hams District Council  Dartmoor National Park  Exmoor National Park  Mid Devon District Council  North Devon District Council | Corporate Communications: Design Service | 2 | 1 |
| Walking Trails In Devon | Devon County Council  Travelwise | *Not stated* | 36 | 10 |
| West Devon Way | Devon County Council  West Devon Borough Council  Dartmoor National Park  South Hams District Council | Graphic Words | 25 | 8 |
