# Supplementary figures and images for: How do brochures encourage walking in natural environments in the UK? A content analysis
Source: Health Promot Int. 2016 Oct 28;33(2):299–310. doi: 10.1093/heapro/daw083 (PMC5892139; doi:10.1093/heapro/daw083)

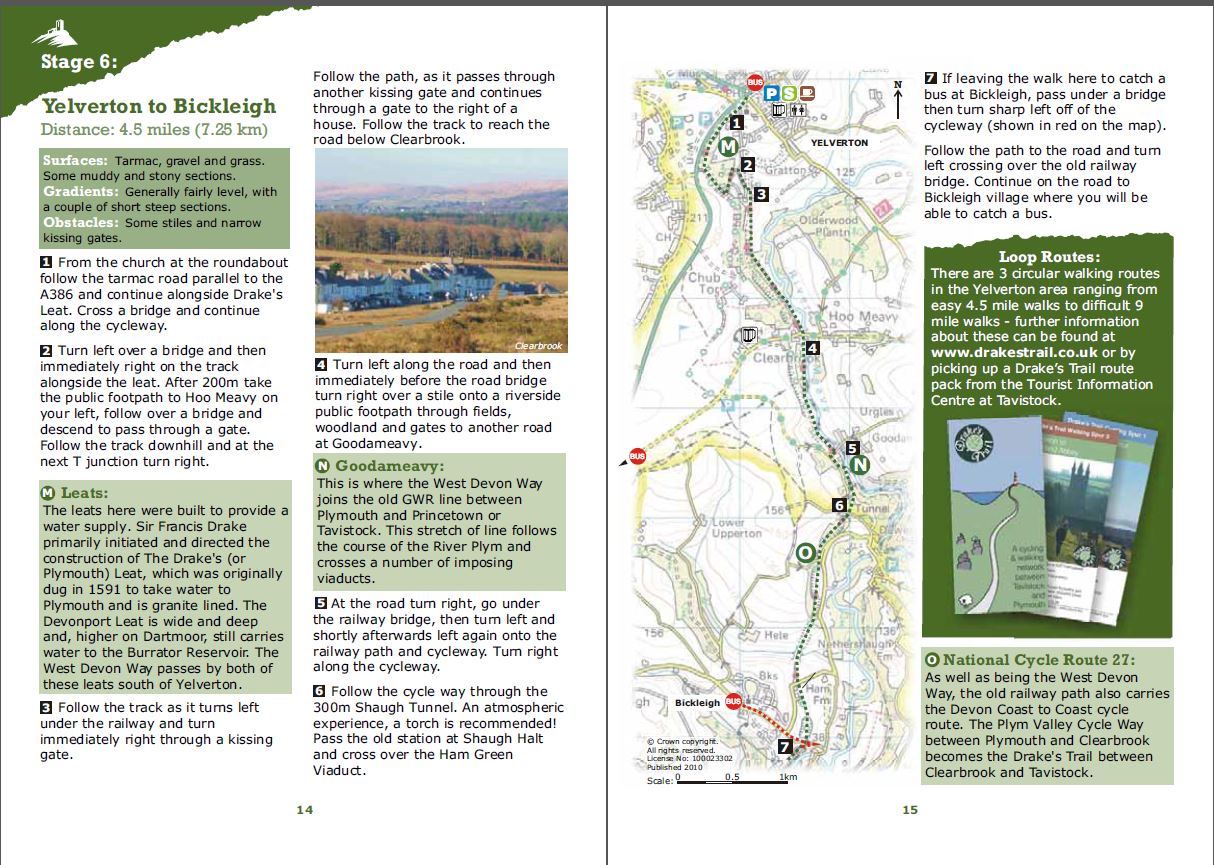

Supplement: Supplementary File S-2 [file daw083_supplementary_file_s-2.jpeg]
