# Supplementary material for: How do brochures encourage walking in natural environments in the UK? A content analysis
Source: Health Promot Int. 2016 Oct 28;33(2):299–310. doi: 10.1093/heapro/daw083 (PMC5892139; doi:10.1093/heapro/daw083)
Supplement: Supplementary File S-3 [file daw083_supplementary_file_s-3.docx]

**Coding Manual**

Broad aims and overview of the coding manual:

The aim of this coding scheme is to facilitate reliable content analysis of text in recreational walking brochures. These are leaflets/booklets/brochures that promote walking routes, often through natural environments, or on national trails, frequently published by city and county councils as well as tourism organisations. Analysis should take place for **all text** included in the main body of the leaflet, and where applicable, pictures and maps will be coded too. Text such as main titles and publishing credits, addresses, phone numbers and website addresses should be categorised as ‘uncoded text’ **unless** these are preceded by text which advises the reader to use these as a means of gathering further information on walking or walking routes. Each category is exclusive (no text can be coded under two categories) and represents a semantic category or concept. Once identified, each category will be counted in order to assess the extent to which each superordinate and subordinate category is represented in the text; used repeatedly in texts; and is represented relative to other super-ordinate and subordinate categories. Category counting will begin from the first page (front cover) of the leaflet as single page leaflets will use this to convey their message.

Coding instructions:

Coders should be aware that the scheme aims to be comprehensive; that is, every piece of text in the brochures should be coded. Anything that it is impossible to categorise should be coded as ‘uncoded text.’ The first thing all coders should do is read through this manual and understand all categories especially the nuances between similar categories. Below are the steps a coder should go through when analysing an individual leaflet:

1. Read the leaflet all the way through twice carefully to get an idea of the common categories that may be included.
2. Re-read and code categories as you go through.
3. Revise step 2, making changes, adding missed instances etc.

Rules and tips for coders:

- A category would begin when the text suggests a semantic category and end only when the text changes semantic meaning to a different category, or changes semantic meaning to uncoded text.
- A **sentence** will be the basic unit of analysis.
  - Most sentences will only include one category (e.g.’ you can see all manner of wildlife including deer, salmon and many species of bird’). This would be one instance of code 20 (despite the list of species) because the semantic meaning does not change. Similarly, lists of route directions in one sentence would only be counted as one instance of code 85.
  - This is unless a sentence is broken by a new category appearance, for example, ‘experts recommend achieving 30 minutes of moderate intensity physical activity on at least 5 days a week and this is surprisingly easy to do.’ The first part of the sentence would be an instance of code 28*,* whereas after the ‘and’ would be code 58*.*
  - In a similar way, the same category **can** appear twice in the same sentence if the sentence is broken by another category appearance. In a sentence such as ‘physical activity can reduce your chances of diabetes, improve your mood and protect against some cancers,’ there is an appearance of physical health outcomes, then mental health outcomes and then physical outcomes again, **this would constitute 2 separate instances** of code 14 and one of code 15*.* Cases such as this appear to be rare.
- One difficult distinction to make is to distinguish between **promotion, encouragement** and **guidance** categories, so this will be made clear here.
  - **Promotion** categories (under the promoting intentions and planning superordinate) are anything that tells the reader to do something without telling them *how* to do it. For example, an instance of code 48 might appear as ‘decide how far along the path you’ll walk.’ It won’t say how to decide (e.g. analyse how far you can walk without getting too tired), or tell you how far to walk (try to walk for 2 miles). ‘Use the waymarkers’ would also be an example of code 51 as it does not tell you how to use the waymarkers.
  - **Encouragement** categories are *confidence building* by saying that it is *easy, simple* or *not very hard* etc to perform a behaviour. For example an instance of code 69 might appear like ‘this 3 mile stretch is an **easy** amble’ as this builds confidence for the reader that the distance goal is easy to complete.
  - **Guidance** categories are *confidence building* by instructing the reader on how to implement a strategy (analyse how far you can walk without getting tired before deciding) or, much more commonly, providing the reader with a direct option. For example, an instance of code 70 might appear like ‘this can be shortened to 9km/5.5 miles by missing Lee Bay and Lee Village.’ This directly provides the reader with a distance goal.
  - The key thing to look out for when a strategy is alluded to is if there is any encouragement or instruction in the sentence; if not, then it will be an example of promoting intentions and planning.

**PROVIDING INFORMATION**

Instances of providing information are when the text provides information about physical activity in general including government recommendations for physical activity **or** when the text provides general information about the advertised walk such as its length, the time it takes to complete the walk etc. “General information” in this instance means text that:

1. **Does not** describe any **benefits** **or outcomes** of physical activity, walking in general or the advertised walk;
2. **Does not** say how **other people** feel about physical activity, walking or the advertised walk;
3. **Does not** promote behavioural or other strategies for physical activity, walking or completing the advertised walk; and
4. **Does not** say that physical activity, walking or the advertised walk is **easy** and does not **encourage or guide** on ways in which to do physical activity, walking or complete the advertised walk.

Instead, it means general statements about physical activity concerning the government’s recommendation of ’30 minutes of moderate intensity physical activity per day on at least 5 days in a week,’ or its variations (e.g. 150 minutes per week). Or it refers to general statements about the walk such as its length, the time it takes to complete it, the state of the terrain (steep, flat, muddy etc), the route (e.g. circular; goes through Budleigh & Exmouth), or amenities on the route (parking, public transport connections, toilets; food outlets), or supplementary map information (e.g. ordinance survey map details). Pictorially, the presence of a map, however good or bad, could also be coded as an instance of providing information. Below are full descriptions of all subordinate categories for providing information.

| **LEVEL 4 CATEGORIES** | **DESCRIPTION** |
| --- | --- |
| 1. Providing information → Recommended physical activity/walking in general → Governmental recommendations → 30 minutes 5 days a week or equivalent  *Information about recommended physical activity guidelines* | Any text which provides information on the Department of Health’s 2004 recommendations for adequate physical activity. At the most basic level, the text must describe the recommendation of 30 minutes of moderate intensity exercise on at least 5 days in a week – or an equivalent of this recommendation. Also count as an instance of this category if the text describes recommended muscle strengthening activity, which is also part of the guidelines. Also count as an instance of this category if the text provides information on recommendations, but it is incorrect (n.b. note instances of incorrect recommendations).  Do not include in this category any text which attempts to build confidence for achieving recommended physical activity rates or provides ways in which recommended levels can be achieved (codes 58 and 59), or describes any outcome of completing such activity (codes 12-17). Also do not include text which states that ‘experts recommend’ or ‘most people walk’ or ‘most people don’t get enough physical activity’ as these are all examples of *Establishing normative beliefs.* |
| 2. Providing information → Advertised walk → Walk characteristics → Distance  *Information about the distance of the advertised route* | Any text which provides information on the distance of the advertised walk or walks. This can be as simple as stating the length of the walk as a number and a distance unit (5 miles), or it can be a description of the length e.g. ‘short walks,’ as long as, in this instance, it is clear that it is describing the length as opposed to the time it takes to complete the walk. If it is unclear whether it is describing the length or the timing, leave the text uncategorised.  Do not include in this category, any text which promotes goal setting based on distances (code 48), describes the distance as easy (code 69), or instructs the reader on a particular distance goal (code 70). |
| 3. Providing information → Advertised walk → Walk characteristics → Timing  *Information about the length it may take to complete the advertised route* | Any text which provides information on the time it takes to complete the walk, or sections of the walk. This can be as simple as stating the time it will take in hours and minutes (1hr 40mins), or it can be more vague such as saying ‘this is a short walk,’ as long as it is clear, in this instance, that ‘short’ refers to the time it takes to complete the walk rather than the distance. If this is ambiguous, leave the text uncategorised.  Do not include in this category, any text which promotes goal setting based on the time of the walk (code 49), describes the time the walk takes as easy (code 71) or instructs the reader on a particular timing-based goal (code 72). |
| 4. Providing information → Advertised walk → Walk characteristics → Terrain  *Information about the terrain of the advertised route* | Any text which provides information on the terrain of the advertised walk or walks. Include in this category, any description of any surface on the route such as steep, flat, smooth, as well as muddy, gravel, off-road etc.  Do not include in this category any text which promotes strategies to overcome difficulties with the terrain (code 53), describes the terrain as easy to manage (code 78), or instructs the reader on ways to overcome difficulties with the terrain (code 79). |
| 5. Providing information → Advertised walk → Walk characteristics → Presence of a map  *Presence of a map* | Any picture or graphic of a map that is related to the advertised walk. The map can be detailed or simplistic, coloured or monochrome, and include the routes on it, or not.  Do not include in this category the presence of a map key (code 50). |
| 6. Providing information → Advertised walk → Walk characteristics → Route  *Information about the overall course of the advertised route* | Any text which provides information on the advertised walk’s overall route. This can include succinct descriptions of the shape of the route (circular, A to B), as well as longer descriptions of what places the route passes through (e.g. ‘one of the walks in this leaflet follows a section of coast path from West Down Beacon back to the station at Exmouth’), or starts/ends at (e.g. ‘starting and finishing at Lower Tamar Lake’). It can also include details about specific features of the route that do not refer to other codes (e.g. ‘there are a number of Geocache boxes hidden along Drake's Trail’).  Do not include in this category, any text which promotes way of overcoming difficulties with, or features of, the route (codes 53-57). Also do not include any text which says that overcoming route difficulties is easy (codes 78, 80, 82, 85 or 86) or instructs the reader on how to overcome aspects of the route that may be challenging (codes 79, 81, 83, 85, or 87). Also do not include in this category any text which refers to signage or waymarkers (codes 51, 73, or 74). |
| 7. Providing information → Advertised walk → Walk characteristics → Map/OS information  *Information about maps related to the advertised route* | Any text which provides information about map materials or Ordinance Survey maps that are related to the advertised walk. The text can simply name a related map or describe a related map and its contents. For example ‘OS Map: 115 Explorer’ would be an instance of this category, but extended details e.g. ‘Ordinance Survey map 115 (Explorer) also covers this route as well as surrounding foot and cycle paths’ would also be an instance.  Do not include in this category any text which describes other *brochures* that have walking routes advertised as this is *maintenance* information (codes 52, 75 or 76). Also do not include in this category, instances of text that promote reading the maps in the brochure as a mean of managing the route (code 55), or tell the reader that reading maps is easy to do (code 82), or instructs the reader on how to read maps (code 83). |
| 8. Providing information → Advertised walk → Amenities → Public Transport  *Information about public transport options related to the advertised route* | Any text which provides information on how the advertised walk can be combined with public transport links. Include in this category instances of where public transport can be used to access the start of the walk, can be supplemented in for part of the walk, or can be used at the end of a walk in order to return to your start point. For example, ‘buses leave Budleigh Salterton from the Public Hall/ Library bus-stop’. Also include text referring to fares/timetables/contact information regarding public transport. Also **include** in this category any text which encourages or guides the reader to take public transport. These **would not** be categorised under enhancing self-efficacy categories as it does not build confidence specifically for the walk/walking.  Do not include in this category text where public transport is part of a map key (code 50). |
| 9. Providing information → Advertised walk → Amenities → Parking  *Information about parking provision related to the advertised route* | Any text which provides information on parking in relation to the advertised walk. Include in this category instances of where the text provides information on the presence of parking facilities at the start of the walk or at other points in the walk where the reader may want to start instead. For example, ‘a car park is available in Topsham on Holman Way that is a short walk from the quay’. Also **include** in this category any text which encourages or guides the reader to use parking facilities. These **would not** be categorised under enhancing self-efficacy categories as they do not build confidence specifically for the walk/walking.  Do not include in this category text where parking facilities are part of a map key (code 50). |
| 10. Providing information → Advertised walk → Amenities → Toilets  *Information about the provision of toilets on the advertised route* | Any text which provides information on the presence of toilets, at any point on the advertised walk. For example, ‘public toilets are beside the route at the top of Phear Park’.  Do not include in this category text where toilets are part of a map key (code 50). |
| 11. Providing information → Advertised walk → Amenities → Refreshments  *Information about refreshments on, or at the end of, the advertised route* | Any text which provides information on café’s, eateries, restaurants and refreshments available on the route or at the destination. For example, ‘the Turf Locks Hotel is a family run pub serving great food, local ales and wines’. The text can be directed at the reader (**you** can choose from a wide variety of restaurants) or not (there is a large selection of restaurants); both examples would be instances of this category. Encouraging or guiding the reader to utilise the eateries would also be counted as instances of this category as these are not building confidence for walking per se and therefore would not be categorised under any enhancing self-efficacy categories. Listed information can also be included here – text such as “Amenities: Café’s, toilets and parking.” The word ‘café’s’ would be an instance of this category (toilets and parking would be instances of the two previous categories).  Do not include in this category text where refreshments are part of a map key (code 50). |

**HIGHLIGHTING CONSEQUENCES**

Instances of highlighting consequences in this coding scheme generally refer to benefits one can acquire from doing physical activity, undertaking walking in general, or undertaking the advertised walk. For physical activity/walking in general, any text which describes a financial (saving money), environmental (less emissions), health (physiological, mental), or social (activity for families/children) benefit should be categorised as an instance of highlighting consequences. As most walking literature refers to outdoor and natural environments, for ‘the advertised walk’ we used categories based on the cultural ecosystems services classifications outlined in the UK National Ecosystem Assessment (UKNEA, 2011). So for the advertised walk, any benefit alluding to monuments/statues, historical/heritage sites, wildlife, views/scenery or botany can be counted as instances of highlighting consequences as well as anything alluding to the benefits families, friends or children can receive, and recreational/leisure facilities at the destination or on the route. **Do not** include as instances of highlighting consequences where the text says **other people** experience similar benefits – these will be counted under **social influence** categories. Below is a list and descriptions of all the subordinate categories for highlighting consequences.

| **LEVEL 4 CATEGORIES** | **DESCRIPTION** |
| --- | --- |
| 12. Highlighting consequences → Recommended physical activity/walking in general → Financial → Cost  *Financial consequences of walking* | Any text which conveys the idea that money saved by choosing physical activity/walking over other modes of transport is a beneficial outcome of walking. The text can either be directed at the reader (walking can save **you** money) or not (walking **saves** money); both examples would be counted as instances of this category. For example, in reference to walking; ‘it’s FREE! No fares, no parking fees, no machine to buy and maintain’.  **Do not** include in this category any text which provides normative information about the financial benefits of physical activity/walking e.g. ‘other people who walk more often, save more money’ (code 29). |
| 13. Highlighting consequences → Recommended physical activity/walking in general → Environmental → Sustainable / Green  *Environmental consequences of walking* | Any text which conveys the idea that physical activity/walking is an ‘environmentally friendly’ option compared to other modes of transport. The text can either be directed at the reader (**you** can help reduce your carbon footprint by choosing to walk over using the car) or not (walking is a better option for the planet); both examples would be counted as instances of this category. For example, ‘Walking is the greenest way to go, with no carbon emissions and no special equipment needed’.  **Do not** include in this category any text which provides normative information about the environmental benefits of physical activity/walking over other modes of transport for example ‘most pedestrians tend to have lower carbon footprints than motorists’ (code 30). |
| 14. Highlighting consequences → Recommended physical activity/walking in general → Health → Physical  *Physical health consequences of walking* | Any text which conveys the idea that physical activity/walking is good for one’s physical health. This can include references to the reduction in risk of certain conditions, improvements in physiological indicators etc. The text can either be directed at the reader (by walking more often **you** can reduce the risk of contracting type 2 diabetes) or not (walking more often results in less chance of contracting type 2 diabetes); both examples would be counted as instances of this category. For example ‘Walking can help prevent gaining weight and helps you to lose weight’.  **Do not** include in this category any text which provides normative information about the physical health benefits of walking over other modes of transport for example ‘people who walk more tend to have less chance of contracting type 2 diabetes’ (code 31). |
| 15. Highlighting consequences → Recommended physical activity/walking in general → Health → Mental  *Mental health consequences of walking* | Any text which conveys the idea that walking is good for one’s mental health. This can include references to the reduction in risk of certain mental health conditions, improvements in mood, stress levels etc. The text can be directed at the reader (increasing **your** walking could make you feel more relaxed) or not (walking can be relaxing); both examples would be counted as instances of this category. For example, ‘walking is invigorating and improves your mood’.  **Do not** include in this category any text which provides normative information about the mental health benefits of walking over other modes of transport for example ‘people who walk more tend to deal with stress better than those who walk less’ (code 32). |
| 16. Highlighting consequences → Recommended physical activity/walking in general → Social → Family / friend / general sociability benefits.  *Social benefits of walking* | Any text which conveys the idea that walking can act as an opportunity to strengthen social bonds with family or friends. This can include references to how the reader may feel better walking in the company of others. The text can be directed at the reader (walking is something **you** may enjoy more if you go with family) or not (walking is more enjoyable with family); both examples would be counted as instances of this category. For example ‘walking can be a very sociable activity’.  **Do not** include in this category any text which provides normative information about the social benefits of walking/being physically active with family or friends such as ‘most people enjoy exercising with their family’ or ‘lots of people tend to go walking with their family at the weekend’ (code 33). Also do not include in this category any text which refers to the *advertised* walk as being suitable for ‘family walks’ or for ‘your children’ etc (code 40). |
| 17. Highlighting consequences → Recommended physical activity/walking in general → Social → Children benefits  *Benefits to children of walking* | Any text which conveys the idea that walking can act as a fun activity for children. This can include references to how the reader’s children may enjoy walking. The text can be directed at the reader; ‘walking is something **your** children may enjoy;’ or not; ‘walking can be an enjoyable activity for children too;’ both examples would be counted as instances of this category.  **Do not** include in this category any text which provides normative information about the social benefits of walking/being physically active with children such as ‘most people like to go for a walk with their children’ or ‘other people enjoy exercise by playing sports and games with their children’ (code 34). Also do not include in this category any text which refers to experiences that children can have on the advertised walk (code 41). |
| 18. Highlighting consequences → Advertised walk → Heritage → Monument  *Viewing a monument as a consequence of walking the advertised route* | Any text which implies that the sight or experience of a monument/statue etc is an outcome of undertaking the walk advertised. The text can either be directed at the reader (and the end of the path **you** can see a war memorial) or not (at the end of the path there is a war memorial); both examples would be counted as instances of this category. Encouraging or guiding the reader to attend to the monument would also be counted as instances of this category as these are not building confidence for walking per se and therefore would not be categorised under any self efficacy categories.  **Do not** include in this category any text which provides normative information about seeing a monument or statue on the advertised walk such as ‘most people tend to pay attention to this monument’ (code 35). |
| 19. Highlighting consequences → Advertised walk → Heritage → Historical site  *Viewing historical points of interest as consequences of walking the advertised route* | Any text which implies that historical points of interest are outcomes of undertaking the walk advertised. The text can either be directed at the reader (in the distance **you** can view Powderham castle) or not (in the distance is Powderham castle); both examples would be counted as instances of this category. Encouraging or guiding the reader to attend to the historical site would also be counted as instances of this category as these are not building confidence for walking per se and therefore would not be categorised under any self efficacy categories.  **Do not** include in this category any text which provides normative information about the Heritage benefits of seeing a historical site on the advertised walk such as ‘most people tend to have a walk around the castle grounds’ (code 36). |
| 20. Highlighting consequences → Advertised walk → Aesthetic → Wildlife  *Viewing wildlife as a consequence of walking the advertised route* | Any text which implies that the sight or experience of wildlife is an outcome of undertaking the walk advertised. The text can either be directed at the reader (**you** can view salmon leaping over this bridge) or not (salmon leap over this bridge in Summer); both examples would be counted as instances of this category. Encouraging or guiding the reader to view the wildlife would also be counted as instances of this category as these are not building confidence for walking per se and therefore would not be categorised under any self efficacy categories.  **Do not** include in this category any text which provides normative information about the aesthetic benefits of seeing wildlife on the advertised walk such as ‘a lot of people like to watch the salmon leap’ (code 37). |
| 21. Highlighting consequences → Advertised walk → Aesthetic → View/scenery  *Viewing scenery as a consequence of walking the advertised route* | Any text which implies that a pleasant view or scene is an outcome of undertaking the walk advertised. The text can either be directed at the reader (and the end of the path **you** can see out over Torbay) or not (at the end of the path there is a great view of Torbay); both examples would be counted as instances of this category. Encouraging or guiding the reader to attend to the scene would also be counted as instances of this category as these are not building confidence for walking per se and therefore would not be categorised under any self efficacy categories.  **Do not** include in this category any text which provides normative information about the aesthetic benefits of viewing scenery on the advertised walk such as ‘a lot of people like to take a rest here and gaze out over the peninsula’ (code 38). |
| 22. Highlighting consequences → Advertised walk → Aesthetic → Botany  *Botanical points of interest as consequences of walking the advertised route* | Any text which implies that a botanical point of interest is an outcome of undertaking the walk advertised. The text can either be directed at the reader (and the end of the path **you** can see a 500 year old tree) or not (at the end of the path there is a 500 year old tree); both examples would be counted as instances of this category. Encouraging or guiding the reader to attend to the scene would also be counted as instances of this category as these are not confidence building for walking per se and therefore would not be categorised under any self efficacy categories.  **Do not** include in this category any text which provides normative information about the aesthetic benefits of botanical points of interest on the advertised walk such as ‘a lot of people like to view these trees in Autumn’ (code 39). |
| 23. Highlighting consequences → Advertised walk → Social → Family or friend benefits  *Social consequences of walking the advertised route* | Any text which implies that the opportunity to strengthen social bonds with family or friends is an outcome of undertaking the walk advertised. The text may for example state that the particular route is best enjoyed as a family, or the destination may be described as a great place to go with friends (implying the reader should undertake the walk with friends). Encouraging or guiding the reader to undertake the walk with friends or family because of the opportunity for heightened enjoyment would also be counted as instances of this category as these are not building confidence for walking per se and therefore would not be categorised under any self efficacy categories.  **Do not** include in this category any text which provides normative information about the social benefits of walking with family or friends on the advertised walk such as ‘most people tend to walk this route with their family’ (code 40). |
| 24. Highlighting consequences → Advertised walk → Social → Children benefits  *Benefits to children of walking the advertised route* | Any text which implies that children will experience enjoyment from partaking in the advertised walk. The text may for example state that particular sections of the walk are perfect for children to run around, or the destination may be described as a great place for children to have fun (implying the reader should take their children with them). Encouraging or guiding the reader to undertake the walk with children because of the opportunities the children would have for heightened enjoyment would also be counted as instances of this category as these are not building confidence for walking per se and therefore would not be categorised under any self efficacy categories.  **Do not** include in this category any text which provides normative information about the social benefits for children on the advertised walk such as ‘most people take their children on this walk due to the mass of play equipment on the route’ (code 41). |
| 25. Highlighting consequences → Advertised walk → Recreational → Accommodation  *Accommodation at the destination as a consequence of walking the advertised route* | Any text which implies that accommodation at the destination (if it is a long walking route) is a desirable outcome of undertaking the advertised walk. The text may for example state that there is a range of hotels at the destination. The text can be directed at the reader (**you** can choose from a wide variety of hotels) or not (there is a large selection of hotels); both examples would be instances of this category. For example, ‘you will discover some wonderful and luxurious self-catering accommodation’. Encouraging or guiding the reader to utilise the accommodation would also be counted as instances of this category as these are not building confidence for walking per se and therefore would not be categorised under any self efficacy categories.  **Do not** include in this category any text which provides normative information about the recreational benefits of utilising accommodation at the end of the advertised walk such as ‘most people choose a guesthouse for an overnight stay’ (code 42). |
| 26. Highlighting consequences → Advertised walk → Recreational → Leisure  *Leisure opportunities as consequences of walking the advertised route* | Any text which implies that other leisure opportunities along the route or at the destination are a desirable outcome of undertaking the advertised walk. The text may for example state that there are shops, cinemas, arcades etc at the destination or that there are play parks along the route. The text can be directed at the reader (**you** could visit one of the many arcades) or not (there are many arcades); both examples would be instances of this category. For example, ‘there are several clubs and training centres available to help you make the most of the Exe’. Encouraging or guiding the reader to utilise leisure opportunities would also be counted as instances of this category as these are not building confidence for walking per se and therefore would not be categorised under any self efficacy categories.  **Do not** include in this category any text which provides normative information about the recreational benefits of utilising leisure facilities on/at the end of the advertised walk such as ‘most people relax at the end of the walk by visiting the cinema or going round the shops’ (code 43). |

**ESTABLISHING NORMATIVE BELIEFS**

Instances of establishing normative beliefs include any text where **people other than the reader** are mentioned in a way that is designed to motivate the reader to undertake physical activity/walking or to undertake the advertised walk. In this sense, almost all the social influence categories are related to, but distinct from, the outcome expectancy categories. For physical activity/walking, instances of establishing normative beliefs can include normative information about government guidelines for physical activity, expert opinion on physical activity, or normative information about financial gains from walking (pedestrians save money), environmental benefits of walking (non-motorists help the planet), or physical or mental health benefits of walking (people who walk more are more healthy/less stressed). For the advertised walk, instances of establishing normative beliefs can include normative information about how others enjoy the same cultural, aesthetic, social and recreational benefits that were detailed in the outcome expectancy categories (e.g. other people enjoy watching the salmon leap; most people take a walk around the castle grounds). A list and descriptions of all subordinate social influence categories is presented below.

| **LEVEL 4 CATEGORIES** | **DESCRIPTION** |
| --- | --- |
| 27. Establishing normative beliefs → Recommended physical activity/walking → Governmental recommendations → Normative information  *Normative information about recommended physical activity guidelines or walking* | Any text which describes other people’s behaviour, attitudes etc in relation to governmental recommendations for physical activity or for walking in general. Text could include statements such as ‘Most people try to do 30 minutes of physical activity per day’ or ‘most people walk daily’ or ‘a lot of people wish to walk more often’ or ‘some people don’t walk at all.’ Virtually any description of other people’s physical activity or walking behaviours can be included in this category.  Do not include in this category information about governmental recommendations for physical activity or information about walking in general that makes no reference to others behaviour such as ‘getting 30 minutes of physical activity per day is ideal’ (code 1). Also do not include in this category text such as ‘the government recommends that everyone achieve 30 minutes of moderate intensity exercise per day’ or ‘experts recommend walking on a daily basis.’ These mention an authority figure and therefore would be categorised under code 28. |
| 28. Establishing normative beliefs → Recommended physical activity/walking → Governmental recommendations → Expert recommendation  *Expert recommendations about physical activity or walking* | Any text which describes an experts’, or any other authority figures’, recommendations about physical activity or about walking in general. Text could include statements such as ‘the UK government suggests that you do 30 minutes of moderate intensity physical activity at least 5 times a week’ or ‘doctors recommend walking as a healthy activity’ or ‘the NHS fully supports walking initiatives.’ Also include in this category, text such as quotes that are attributed to an authority for example ‘”walking is good for you” – Chief Medical Officer.’  Do not include in this category any text which only refers to ‘most’ people or ‘some’ people. When the subject of the sentence is not an authority, the text should be categorised under code 27. Information about recommendations that does not mention an expert or authority (code 1). |
| 29. Establishing normative beliefs → Recommended physical activity/walking → Financial→ Normative information  *Normative information about the financial consequences of walking* | Any text which describes the financial benefits others experience by choosing to walk/be physically active over other modes of transport. Text could include statements such as ‘almost all pedestrians save money by walking rather than taking a car’ or ‘people who save money by walking spend it on things they enjoy.’  Note that these statements would **not** be instances of code 12, because although they convey a benefit, semantically, they provide normative information. An example of code 12 could be ‘walking can save you money’. |
| 30. Establishing normative beliefs → Recommended physical activity/walking → Environmental → Normative information  *Normative information about the environmental consequences of walking* | Any text which describes the environmental benefits others get by choosing to walk/be physically active over other modes of transport. Text could include statements such as ‘people who walk more often have a lower carbon footprint’ or ‘people who walk or cycle put less pressure on the planet.’  Note that these statements would **not** be coded under code 13, because although they convey a benefit, semantically, they provide normative information. An example of code 13 could be ‘walking is better than driving for the planet’. |
| 31. Establishing normative beliefs → Recommended physical activity/walking → Health → Normative information – physical  *Normative information about the physical health consequences of walking* | Any text which describes the physical health benefits others get by choosing to walk/be physically active over other modes of transport. Text could include statements such as ‘people who exercise more are healthier’ or ‘people who walk every day are at less risk of getting heart problems.’  Note that these statements would **not** be coded under code 14, because although they convey a benefit, semantically, they provide normative information. An example of code 14 could be ‘walking is good for your heart’. |
| 32. Establishing normative beliefs → Recommended physical activity/walking → Health → Normative information – mental  *Normative information about the mental health consequences of walking* | Any text which describes the mental health benefits others get by choosing to walk/be physically active over other modes of transport. Text could include statements such as ‘others who exercise regularly are generally more relaxed’ or ‘most people who walk every day are less stressed.’  Note that these statements would **not** be coded under code 14, because although they convey a benefit, semantically, they provide normative information. An example of code 15 could be ‘walking can help you relax’. |
| 33. Establishing normative beliefs → Recommended physical activity/walking → Social → Normative information - family or friend benefits  *Normative information about the social consequences of walking* | Any text which describes the enjoyment others get from undertaking physical activity/walking with family or friends. Text could include statements such as ‘most people enjoy walking with friends’ or ‘a lot of people choose to undertake exercise with their family.’  Note that these statements would **not** be coded under code 16, because although they convey a benefit, semantically, they provide normative information. An example of code 16 could be ‘walking can be very sociable’. |
| 34. Establishing normative beliefs → Recommended physical activity/walking → Social → Normative information - children benefits  *Normative information about the benefits to children of walking* | Any text which describes the enjoyment other may get from undertaking physical activity/walking with children. Text could include statements such as ‘a lot of people get their daily exercise through playing sports or games with their children’ or ‘most people enjoy walking with their children.’  Note that these statements would **not** be coded under code 17, because although they convey a benefit, semantically, they provide normative information. An example of code 17 could be ‘walking can be fun for your children’. |
| 35. Establishing normative beliefs → The advertised walk → Heritage→ NI monument  *Normative information about viewing a monument on the advertised route* | Any text which describes a monument/statue etc as being a benefit of the advertised walk that other people enjoy. Text could include statements such as ‘most people like to take a minute to look at this monument.’  Note that this sort of statement would **not** be coded under code 18, because although they convey a benefit, semantically, they provide normative information. An example of code 18 could be ‘there is a war memorial at the end of the path’. |
| 36. Establishing normative beliefs → The advertised walk → Heritage→ NI historical site  *Normative information about viewing historical points of interest on the advertised route* | Any text which describes a historical site as being a benefit of the advertised walk that other people enjoy. Text could include statements such as ‘a lot of people tend to take time out from the main route to walk around the castle grounds.’  Note that this would **not** be coded under code 19, because although they convey a benefit, semantically, they provide normative information. An example of code 19 could be ‘the castle was built in 1800’. |
| 37. Establishing normative beliefs → The advertised walk → Aesthetic→ NI wildlife  *Normative information about viewing wildlife on the advertised route* | Any text which describes wildlife as being a benefit of the advertised walk that other people enjoy. Text could include statements such as ‘people like to stop in summer and try and catch a glimpse of the salmon leaping.’  Note that this would **not** be coded under code 20, because although they convey a benefit, semantically, they provide normative information. An example of code 20 could be ‘you can see salmon leaping here’. |
| 38. Establishing normative beliefs → The advertised walk → Aesthetic→ NI view/scenery  *Normative information about viewing scenery on the advertised route* | Any text which describes views or scenery as being a benefit of the advertised walk that other people enjoy. Text could include statements such as ‘people like to take a breather and look out across the peninsular here’ or ‘often people will sit at the top of the cliff and look out to sea.’  Note that this would **not** be coded under code 21, because although they convey a benefit, semantically, they provide normative information. An example of code 21 could be ‘there are great views over the Exe here’. |
| 39. Establishing normative beliefs → The advertised walk → Aesthetic→ NI botany  *Normative information about viewing botanical points of interest on the advertised route* | Any text which describes seeing botanical points of interest as being a benefit of the advertised walk that others enjoy. Text could include statements such as ‘some people like to gaze at the tree which is over 1000 years old.’  Note that this would **not** be coded under code 22, because although they convey a benefit, semantically, they provide normative information. An example of code 22 could be ‘behind is an attractive landscape of salt marshes, now managed as a nature reserve’. |
| 40. Establishing normative beliefs → The advertised walk → Social → NI family or friend benefits  *Normative information about the social consequences of walking the advertised route* | Any text which describes the opportunity to strengthen social bonds with friends or family as a benefit of the advertised walk that other people enjoy. Text could include statements such as ‘most people tend to walk this route with their family’ or ‘lots of people enjoy this walk with friends.’  Note that these would **not** be coded under code 23, because although they convey a benefit, semantically, they provide normative information. An example of code 23 could be ‘this walk is for all the family to enjoy’. |
| 41. Establishing normative beliefs → The advertised walk → Social → NI children benefits  *Normative information about the benefits to children of walking the advertised route* | Any text which describes the idea that children enjoy the walk as a benefit of the advertised walk that other people enjoy. Text could include statements such as ‘most people love to see their children running across the fields’ or ‘other families get a real kick out of the freedom their children have to explore the natural environment around them.’  Note that these would **not** be coded under code 24, because although they convey a benefit, semantically, they provide normative information. An example of code 24 could be ‘for children there are quizzes and puzzles to solve along the way’. |
| 42. Establishing normative beliefs → The advertised walk → Recreational → NI accommodation  *Normative information about accommodation at the destination of the advertised route* | Any text which describes accommodation at the destination (if it is a long walking route) as a benefit of undertaking the advertised walk that others enjoy. Text could include statements such as ‘most guests tend to stay in a B&B’ or ‘a lot of people choose one of the smaller hotels.’  Note that these would **not** be coded under code 25, because although they convey a benefit, semantically, they provide normative information. An example of code 25 could be ‘delightful hotels on the coast or in the country, cosy country pubs, idyllic guesthouses and farms, ideal for family holidays’. |
| 43. Establishing normative beliefs → The advertised walk → Recreational → NI leisure  *Normative information about leisure opportunities on, or at the end of, the advertised route* | Any text which describes leisure facilities on route or at the destination as a benefit of the advertised walk that other people enjoy. Text could include statements such as ‘a lot of people make the most of the destination by going to the cinema or doing some shopping’ or ‘at the end of the walk, most people like to visit the swimming pool or arcade.’  Note that these would **not** be coded under code 26, because although they convey a benefit, semantically, they provide normative information. An example of code 26 could be ‘sandy beaches and water based recreation are some of the attractions to be found on the Exe Estuary’. |

**PROMOTING INTENTIONS AND PLANNING**

Instances of promoting intentions and planning categories can be any text which aims to promote a behavioural strategy for undertaking physical activity, walking in general, or for completing the advertised walk **without encouraging** (saying that the technique will make the intended behaviour easier) **or guiding** (telling the reader how to utilise the technique or what, for example, goals, should be set) the reader about these strategies. For example, text which advises the reader to try and set distance (or amount of steps) goals, or set aside time for walking/physical activity or asks them to ‘try walking regularly’ would be instances of promoting intentions for physical activity or walking in general. Text which asks the reader to ‘think about breaking the advertised walk up,’ or ‘consider how long they will walk before resting,’ or advises them to ‘follow waymarkers’ or ‘access other walk information’ would also be instances of promoting intentions as they do not provide information on *how* to do these things. In the case of the advertised walk, it can also include strategies about how to overcome difficulties with the walk such as inviting the reader to take waterproofs, look out for muddy patches, think about map reading, or tells them to consider following the printed directions. Promotion of general barrier reduction e.g. ‘don’t worry about the weather’ (where weather is common barrier) is also included in promoting intentions and planning. Pictorially, the presence of a map key can also be counted as an instance of promoting intentions and planning. A list and descriptions of all subordinate categories for promoting intentions and planning is displayed below.

| **LEVEL 4 CATEGORIES** | **DESCRIPTION** |
| --- | --- |
| 44. Promoting intentions and planning → Recommended physical activity/walking → Walk management → Distance or route based goals  *Prompting walking goals based on distance* | Any text which tells the reader to set distance or route based goals for physical activity or walking in general **without** guiding them on how to do that **or** telling them that this will make achieving those aims easier. For example, ‘decide how far you will walk’ or ‘plan a walk that is suitable for your level of fitness’. In the latter, this would be as long as the text is referring to distance rather than time. It could even be more general such as ‘try walking further’ or ‘go on longer-distance walks;’ again these refer to distance goals without telling the reader how to achieve them.  Do not include in this category any text which suggests that setting goals for walking/physical activity makes achieving those things easier such as ‘it is easy to walk long distances’ or ‘sticking to a walking aim is easy’ (code 60). Also do not include in this category any text which guides the reader on how to set goals or what goals to set such as ‘write on your calendar how far you will walk on a particular day, then stick to it’ or ‘attempt to walk around where you live every day’ (code 61). Also do not include in this category text which promotes distance goals for the advertised walk (code 48). |
| 45. Promoting intentions and planning → Recommended physical activity/walking → Walk management → Time based goals  *Prompting walking goals based on time* | Any text which tells the reader to manage their time for physical activity or walking in general **without** telling them precisely how to do that **or** telling them that this strategy will make achieving those aims easier. Text could include statements such as ‘set aside some time for exercise’ or ‘consider freeing up some time for walking’ or ‘why not try walking on a weekly basis?’ It could be even more general such as ‘walk regularly’ or ‘walk more often;’ these still refer to time based goals without telling the reader how to achieve them.  Do not include in this category any text which suggests that time based goals are easy or makes physical activity/walking aims easier to achieve such as ‘finding an hour a day to walk is easy’ or ‘setting aside an evening is an easy way of knowing when you will exercise’ (code 62). Also do not include text which guides the reader on what time based goals to set such as ‘set aside an hour in your evening and use it to walk around where you live’ or ‘make sure that at the weekend you have a whole afternoon free so that you can go out to your local environments and get some exercise’ (code 63). Also do not include in this category text which promotes time based goals for the advertised walk (code 49). |
| 46. Promoting intentions and planning → Recommended physical activity/walking → Walk management → Barrier reduction  *Prompting barrier reduction for walking* | Any text which invites the reader to reduce general barriers to completing recommended physical activity or walking in general **without** telling them how to overcome these barriers **or** saying that overcoming these barriers is easy. Text can include statements such as ‘you can even walk in the rain’ or ‘walking need not require special equipment.’ These address common barriers (weather, provision of correct equipment) but do not tell the reader how to, for example, walk in the rain, or if there are circumstances where equipment is required.  Do not include in this category any text which says that overcoming general barriers to achieving recommended physical activity/walking in general is easy such as ‘walking in bad weather is rather simple’ or ‘getting yourself motivated to do exercise isn’t difficult’ (code 64). Also do not include in this category any text which guides the reader upon how to overcome barriers such as ‘have a healthy meal beforehand’ or ‘you can start slowly and build up gradually’ (code 65). Also do not include in this category text which promotes overcoming barriers related to the advertised walk (code 57). |
| 47. Promoting intentions and planning → Recommended physical activity/walking → Reinforcement → Maintenance  *Prompting repeated walking* | Any text which tells the reader to keep walking or keep practicing physical activity **without** telling the reader how to do this **or** saying that this is easy. Text could include statements such as ‘make sure you walk every day’ or ‘once you’ve started walking, keep it up’ or ‘why not go walking every day?’  Do not include in this category any text which suggests that continuing walking or physical activity behaviours is easy such as ‘it is easy to keep up walking’ or ‘exercising often need not be difficult’ (code 66). Also do not include in this category text which guides the reader on how to continue walking/physical activity behaviours such as ‘keep exercising regularly by planning what you will do and when’ (code 67). Also do not include in this category text which promotes continued walking or exercising that is related to the advertised walk, such as the promotion of other brochures, walking routes etc (code 52). |
| 48. Promoting intentions and planning → The advertised walk → Walk management → Distance or route based goals  *Prompting distance goals for the advertised route* | Any text which tells the reader to set distance or route based goals for the advertised walk **without** saying that setting goals for the advertised walk is easy **or** guiding the reader on how to set goals/what goals to set for the advertised walk. For example, ‘as well as circular routes around the Exe, there are also several long distance routes which can be met on the estuary’ or ‘perhaps try a slightly longer walk of some 21 (4 km) which passes more of the Tamar Lakes.’  Do not include in this category any text that suggests that setting goals for the advertised walk is easy such as ‘it is easy to divide the walk into more manageable stretches’ or ‘the route is a lot easier if done in stages’ (code 69). Also do not include in this category text which guides the reader on how to set distance goals for the advertised walk or what goals to set such as ‘you can shorten the walk by 1.9 kilometres (1.2 miles) by following the East Devon Way’ (code 70). |
| 49. Promoting intentions and planning → The advertised walk → Walk management → Time based goals  *Prompting time goals for the advertised route* | Any text which tells the reader to set time based goals for the advertised walk **without** saying that time based goals for the advertised walk are easy **or** guiding the reader on how to manage their time for the advertised walk. For example ‘many of the trails link up to form a network of walks, providing opportunities to do a shorter day or half day walk.’  Do not include in this category any text that suggests that setting time based goals for the advertised walk is easy such as ‘the walk only takes an hour so you can fit it in your day with ease’ (code 71). Also do not include in this category text which guides the reader on how to set time goals for the advertised walk or what time goals to set such as ‘the Exe Valley Way can be divided up into a series of 10 stages, most of which can be walked comfortably by most walkers in half a day’ (code 72). |
| 50. Promoting intentions and planning → The advertised walk → Walk management → Map key  *Presence of a map key* | Any picture/diagram that is a key to a map. Do not code if it is not obvious to which map the key refers. |
| 51. Promoting intentions and planning → The advertised walk → Reinforcement → Stimulus control  *Prompting attention to signage on the advertised route* | Any text which tells the reader to attend or ‘look out for’ discriminative stimuli on the advertised walk **without** saying that this would make the walk easier **or** precisely guiding how they should use the stimuli. Discriminative stimuli in this instance would mean signage or waymarkers that reinforce further or continued walking. Text could include statements such as ‘use the waymarkers’ or ‘follow the coastal footpath signs.’  Do not include in this category text which suggests that using signage/waymarkers makes the walk easier such as ‘it is easy to follow the signs’ or ‘it is easy to keep track of how far you’ve walked by looking at the waymarkers’ (code 73). Also do not include in this category text which guides the reader on how to use the waymarkers/signage such as ‘look at each waymarker to see how far you have walked and how far you have to go’ or ‘as you pass each waymarker, look closely to make sure you are following the right path’ (code 74). |
| 52. Promoting intentions and planning → The advertised walk → Reinforcement → Maintenance  *Prompting repeated recreational walking similar to the advertised route* | Any text which promotes a way in which the reader can continue to walk routes similar to the advertised walk. This could mean accessing anything related to the advertised walk that promotes continued walking in a similar fashion such as looking up other related leaflets. Text could include statements such as ‘look up our other walks’ or ‘download more brochures in this series’ or ‘a small book is available, containing information and suggested routes, price £2.99.’  Do not include in this category any text which suggests that accessing related walk information is easy such as ‘it is simple to download the other brochures in this series’ or that it is easy to continue doing similar walks ‘there are so many walks in Devon, it is easy to choose one to do every week’ (code 75). Also do not include in this category text which guides the reader on how to access related walk information such as ‘this is one of the many walks that can be found at www.southwestcoastpath.com’ (code 76). |
| 53. Promoting intentions and planning → The advertised walk → Route difficulties → Terrain management  *Prompting ways to overcome difficulties with the terrain on the advertised route* | Any text which tells the reader to be wary/alert to the terrain of the advertised walk **without** saying that it is easy to cope with the terrain **or** guiding on ways to overcome difficulties with the terrain. For example ‘be aware of the dangers from rising tides, soft mud, cliff edges and strong currents.’  Do not include in this category any text which states that it is easy to cope with difficulties in the terrain such as ‘although muddy, the walk is generally easy’ (code 78). Also do not include in this category any text which guides on how to overcome difficulties with the terrain such as ‘do not attempt the walk via the beach for two hours either side of high tide’ or ‘this route is closed during the shooting season from 1st October to 1st February, and walkers should follow the alternative route along the quiet road instead at that time’ (code 79). |
| 54. Promoting intentions and planning → The advertised walk → Route difficulties → Appropriate equipment  *Prompting equipment needed for walking the advertised route* | Any text which tells the reader to consider appropriate equipment **without** telling them that it will make the walk easier **or** guiding them to directly take appropriate equipment. Text could include statements such as ‘consider layering up your clothes.’  Do not include in this category any text which states that the walk is easier with appropriate equipment/clothing such as ‘having walking boots will make for an easier trek’ (code 80). Also do not include in this category any text which guides the reader directly to take appropriate equipment or wear certain clothing such as ‘dress according to the conditions, and take water with you even on a cloudy day’ or ‘but don’t rely on them (cafés) for your refreshments – always take more than you think you will need’ (code 81). |
| 55. Promoting intentions and planning → The advertised walk → Route difficulties → Map reading  *Prompting map reading for the advertised route* | Any text which tells the reader to read the accompanying map **without** saying that this will make the walk easier **or** guiding them on how to read the map efficiently. For example ‘the route should be followed with the help of the sketch maps inside this booklet.’  Do not include in this category any text which states that the walk would be made easier by reading the map such as ‘the map is clearly labelled so you can follow the route with ease’ (code 82). Also do not include in this category text which tells the reader how to use the map such as ‘the map can be used to create your own trails depending on where you would like to visit, your means of transport, and how long you would like to take’ (code 83). |
| 56. Promoting intentions and planning → The advertised walk → Route difficulties → Direction taking  *Prompting direction taking for the advertised route* | Any text which tells the reader to follow directions **without** telling them that this will make the walk easier **or** specifically outlining directions. Text could include statement such as ‘follow the directions’ or ‘consider the steps outlined in this leaflet.’ Text could also refer to the direction of the route more generally such as ‘follow the coastline.’  Do not include in this category text which tells the reader that the walk will be easier if they follow directions such as ‘following the steps in this booklet will make navigating the route a lot easier’ or text that says the walk is easy due to its overall direction such as ‘the circularity of this walk makes it easy to follow’ (code 84). Also do not include in this category text which outlines directions for the reader such as ‘leaving Polsloe Bridge station, cross the main Pinhoe Road and go under the railway bridge’ (code 85). |
| 57. Promoting intentions and planning → The advertised walk → Route difficulties → Barrier reduction  *Prompting barrier reduction on the advertised route* | Any text which tells the reader to undertake the walk in spite of potential barriers, but **does not** tell them how to overcome such barriers **or** that adjusting to such barriers is easy. Text could include statements such as ‘it is also suitable for use by cyclists, wheelchair users or as a pushchair walk’ as this reduces disability/children related barriers. Another example could be ‘the walks are not competitive – so it doesn’t matter if you are not as fast as anyone else’ as this reduces speed/pace related barriers. However, in neither case does the text say that, for example, the path is **easy** for wheelchairs/pushchairs/at a slow pace, nor does the text instruct on how to best manage the walk with a wheelchair/pushchair/slower pace.  Do not include in this category text which states that barriers to the advertised walk are easy to overcome such as ‘even in wet weather, this route is easy to do’ (code 86). Also do not include in this category text which guides the reader on how to overcome general barriers to the advertised walk such as ‘buses on A376 Exmouth Road or trains from Lympstone can be used to shorten the walk if required’ (code 87). This would be categorised under code 87 as it refers to a general barrier (access via public transport) rather than one of the other stated barriers in this coding scheme (e.g. terrain, equipment, map reading, direction taking). |

**ENHANCING SELF-EFFICACY**

Instances of enhancing self-efficacy categories can be any text which **builds confidence** in the reader for doing physical activity, walking in general, or builds confidence for undertaking the advertised walk. It can be text which **encourages** the reader to utilise a behavioural strategy for doing physical activity/walking in general by saying it is easy to set distance or time goals or to continue doing physical activity. It can also include general encouragement for completing government recommended levels of physical activity (e.g. it is easy to do 30 minutes per day). Self-efficacy text can also guide on these strategies for example by saying what goals the reader should set and/or how to set them (e.g. reserve an hour every weekday evening to walk around some fields; write on your calendar when you will do your 30 minutes of exercise). For the advertised walk, text can similarly encourage the reader that setting distance or time goals, continuing similar walks, or following waymarkers (discriminative stimuli that reinforce walking) is easy to do as well as say that managing the terrain, reading maps, following directions, or reducing general barriers (access weather) to doing the advertised walk is simple. Again, self-efficacy text can also guide on these things, for example how the reader can upkeep walking by telling them how to access related walk information (e.g. go to *website* and download the electronic versions of the other walks in this series and keep them in a folder on your computer so you can access them at any time). Importantly, self-efficacy text can guide on direction taking (e.g. turn left at the end of Bonhay Road, then cross the river) which builds confidence in the reader that they can follow the correct route. Instances of this (guidance for direction taking) are perhaps the most commonly used strategy in walking leaflets. Pictorially, modelling walking behaviours (generally, or for the advertised walk) would also be instances of enhancing self-efficacy categories. A list and descriptions of all the subordinate self-efficacy categories is listed below.

| **LEVEL 4 CATEGORIES** | **DESCRIPTION** |
| --- | --- |
| 58. Enhancing self-efficacy → Recommended physical activity/walking → Governmental recommendations → Encouraging recommended physical activity/walking  *Encouraging recommended levels of physical activity* | Any text which conveys the sense that achieving recommended levels of walking is **easy** for the reader to do. This can include text that states for example ‘getting 30 minutes 5 days a week is easy.’  Do not include in this category text which guides the reader on means by which they can achieve recommended levels of walking – any instance of this should be categorised beneath one of the guidance categories below. Also do not include any text which states that managing time for walking, or setting goals for walking, is easy. These should be categorised under the relevant encouragement categories below. |
| 59. Enhancing self-efficacy → Recommended physical activity/walking → Governmental recommendations → Guidance for recommended physical activity/walking  *Guidance on how to achieve recommended levels of physical activity* | Any text which guides the reader on how to achieve recommended levels of physical activity, or how to undertake walking in general, without reference to distance or time based goals, general barrier reduction strategy, or maintenance strategies. For example ‘Doing 10,000 steps per day will contribute to the recommendation of moderate-intensity physical activity for at least 30 minutes on 5 or more days per week.’ This statement does not refer to distance or times goals, maintenance strategies or barriers (e.g. weather) but provides guidance on other ways (in this case, step counts) of achieving physical activity.  Do not include in this category text which merely states that it is easy to achieve recommended amounts of physical activity or walking in general (code 58). |
| 60. Enhancing self-efficacy → Recommended physical activity/walking → Walk management → Encouraging distance or route based goals  *Encouraging walking goals based on distances* | Any text which conveys the sense that distance or route based goals for recommended levels of physical activity/walking in general are **easy** for the reader to do **without** guiding the reader on how to do it. This can include text for example that states ’it is **easy** to walk 2 miles every day.’  Do not include in this category text which guides the reader on how to set a distance goal e.g. ‘mark on your calendar how far you’ll walk each day’; or what distance goals to set ‘walk 2 miles around your village every day’ (code 61). |
| 61. Enhancing self-efficacy → Recommended physical activity/walking → Walk management → Guidance for distance or route based goals  *Guidance on walking goals based on distances* | Any text which guides the reader on how to set distance/route goals, or more commonly, on how to achieve distance/route goals, for recommended levels of physical activity/walking in general. For example, ‘walk around your local area for 2 miles every day; you could do this by walking your children to school and back.’  Do not include in this category text which states the ease with which one can achieve a distance/route goal (code 60). Also do not include text which merely promotes distance goals such as ‘try walking longer distances around your village’ (code 44). |
| 62. Enhancing self-efficacy → Recommended physical activity/walking → Walk management → Encouraging time based goals  *Encouraging walking goals based on time* | Any text which conveys the sense that setting time based goals for recommended levels of physical activity/walking in general is **easy** for the reader to do **without** guiding the reader on how to do it. This can include text for example that states ‘it is easy to fit daily walking into your everyday routine’ or ‘finding time in your day to do some walking is not difficult.’  Do not include in this category text which guides the reader on how to set time based goals or what time based goals to set e.g. ‘by walking to work instead of driving, you will dramatically increase the time you spend walking each day’ (code 63). |
| 63. Enhancing self-efficacy → Recommended physical activity/walking → Walk management → Guidance for time based goals  *Guidance on walking goals based on time* | Any text which guides the reader on how to set time based goals, or more commonly, tells the reader to manage their time in a particular way for the purposes of achieving recommended levels of physical activity/walking in general. For example, ‘replace an hour of TV in the evening with an hour of physical activity such as going for a walk.’  Do not include in this category text which states the ease with which one can set time based goals (code 62). Also do not include in this category text which merely promotes time based goals e.g. ‘free up some time exercise’ (code 45). |
| 64. Enhancing self-efficacy → Recommended physical activity/walking → Walk management → Encouraging barrier reduction  *Encouraging the reduction of barriers to walking* | Any text that says that overcoming general barriers to physical activity or walking in general is easy. Text could include statements such as ‘getting motivated for walking is easy’ or ‘you shouldn’t worry about the bad weather, even walking in the rain is pretty simple’. These are all examples of ways of encouraging physical activity or walking through reducing traditional barriers but don’t refer to distance/time based goals or maintenance.  Do not include in this category text which guides the reader on how to overcome traditional barriers to physical activity/walking in general, such as ‘have an energy filled snack like a banana an hour before exercising’ (code 65). |
| 65. Enhancing self-efficacy → Recommended physical activity/walking → Walk management → Guidance for barrier reduction  *Guidance on the reduction of barriers to walking* | Any text that guides the reader on how to overcome general barriers to physical activity or walking in general. Text could include statements such as ‘prepare yourself for walking by having a quick energy boosting snack’ or ‘if you do not have the time or energy to walk all the way to your destination, walking combined with buses or trains is a good way to get there.’ This is an example of guiding the reader on ways to do physical activity without reference to distance /time based goals or maintenance strategies, but through reducing general barriers (motivation/amount of energy).  Do not include in this category and text which states that general reduction of barriers to physical activity/walking in general is easy (code 64). Also do not include in this category text that merely tells the reader to reduce barriers e.g. ‘you can even walk in the rain’ or ‘get yourself motivated before exercise’ (code 46). |
| 66. Enhancing self-efficacy → Recommended physical activity/walking → Reinforcement → Encouraging maintenance  *Encouraging repeated walking* | Any text which conveys the sense that continuing practicing physical activity/walking in general is **easy** for the reader to do **without** guiding the reader on how to do it. For example, ‘it is easy to keep up a walking routine.”  Do not include in this category text which guides the reader upon how to continue physical activity/walking in general such as ‘making sure all short leisure trips are taken by foot is a good way of continuing to walk more’ (code 67). |
| 67. Enhancing self-efficacy → Recommended physical activity/walking → Reinforcement → Guidance for maintenance  *Guidance on ways to continue walking* | Any text which guides the reader on ways to continue walking or practicing physical activity in general. For example, ‘committing with a friend to going for a short run every week is a good way of keeping up a physical activity routine.’  Do not include in this category text which states the ease with which one can continue walking/practicing physical activity (code 66). Also do not include in this category any text which merely promotes maintenance behaviours such as ‘try walking every day’ or ‘think about exercising regularly’ (code 47). |
| 68. Enhancing self-efficacy → Recommended physical activity/walking → Reinforcement → Modelling  *Modelling physical activity pictorially* | Any picture of people walking which **is not** related to the advertised walk. For example somebody walking down a nondescript street.  Do not include in this category a picture of someone walking a part of the advertised walk (code 77). |
| 69. Enhancing self-efficacy → The advertised walk →Walk management → Encouraging distance or route based goals  *Encouraging distance walking goals for the advertised route* | Any text which conveys the sense that setting distance or route based goals for the advertised walk is **easy**. This can include anything which says the walk is ‘easy to break up’ or ‘easy to divide into stages’ for example, **without** guiding the reader on specific ways on how to do so.  Do not include in this category text which provides distance or route based goals for the advertised walk such as ‘this can be shortened to 9km/5.5 miles by missing Lee Bay and Lee Village and to 7km/4.5 miles by missing Morte Point’ (code 70). |
| 70. Enhancing self-efficacy → The advertised walk → Walk management → Guidance for distance or route based goals  *Guidance on distance walking goals for the advertised route* | Any text which guides the reader on how to set distance or route based goals for the advertised walk or, more usually, provides the reader with options on how to ‘break up’ the walk into more manageable sections or stages such as ‘you can shorten the walk by 1.9 kilometres (1.2 miles) by following the East Devon Way.’  Do not include in this category text which only says that distance goals for the advertised walk are easy to set (code 69). Also do not include in this category text which merely promotes distance goals for the advertise walk such as ‘try breaking up the walk into stages’ (code 48). |
| 71. Enhancing self-efficacy → The advertised walk → Walk management → Encouraging time based goals  *Encouraging timed walking goals for the advertised route* | Any text which conveys the sense that setting time based goals for the advertised walk is an **easy** thing to do **without** guiding the reader on how to set time based goals such as ‘all the walks in this leaflet can easily be incorporated into your daily routine.’  Do not include in this category any text which tells the reader which guides the reader on what time based goals to set, such as ‘the Exe Valley Way can be divided up into a series of 10 stages, most of which can be walked comfortably by most walkers in half a day’ (code 72). |
| 72. Enhancing self-efficacy → The advertised walk → Walk management→ Guidance for time based goals  *Guidance on timed walking goals for the advertised route* | Any text which guides the reader on how to set time based goals or what time based goals to set such as ‘though an active rambler may be able to complete the walk in a few days, the majority will need longer, even a full fortnight’ or ‘the route can be divided up in to a series of 10 stages, most of which can be walked in half a day.’  Do not include in this category text which only tells the reader that it is easy to manage timing of the walks (code 71). Also do not include in this category text which merely promotes time based goals for the advertised walk (without guidance on what goals to set) such as ‘there are opportunities to do a shorter day or half day walk’ (code 49). |
| 73. Enhancing self-efficacy → The advertised walk → Reinforcement → Encouraging stimulus control  *Encouraging attention to signage on the advertised route* | Any text which conveys the sense that using discriminative stimuli is **easy** or any text that suggests that discriminative stimuli make walking easier **without** guiding the reader to directly use or attend to discriminative stimuli. Discriminative stimuli in this instance tend to mean anything along the route that encourages further walking – most commonly waymarkers or other signage. This could include text which states that it is easy to ‘stay on track as there are waymarkers throughout’ or text that says ‘it is easy to follow the signs.’  Do not include in this category text which guides the reader on how to use signage/waymarkers such as ‘keep track of how far you’ve walked using the waymarkers’ (code 74). |
| 74. Enhancing self-efficacy → The advertised walk → Reinforcement → Guidance for stimulus control  *Guidance on attending to signage on the advertised route* | Any text which guides the reader on how to attend to discriminative stimuli on the route. An example of this could be ‘use the waymarkers to tell how far you’ve travelled and how far you have to go’ or ‘pay attention to the distance markings on the signs so you can keep track of how far you’ve walked.’  Do not include in this category text which only states that discriminative stimuli (signage) make the walk easier or any text that only states it is easy to use the signage (code 73). Also do not include in this category text which merely promotes the use of waymarkers or signage e.g. ‘keep an eye open for the mauve arrows marking the East Devon Way’ (code 51). |
| 75. Enhancing self-efficacy → The advertised walk → Reinforcement → Encouraging maintenance  *Encouraging repeated recreational walks similar to the advertised route* | Any text which conveys the **ease** with which one can continue to walk routes related to the advertised walk **without** guiding the reader on how to access other similar walks such as ‘accessing the other walks in this series couldn’t be easier.’  Do not include in this category any text which guides the reader on how to access further information related to similar walks such as ‘Leaflets are available from TIC, our website: www.exe-estuary.org,or by contacting us via email: exeestua@devon.gov.uk’ (code 76). |
| 76. Enhancing self-efficacy → The advertised walk → Reinforcement → Guidance for maintenance  *Guidance on repeated recreational walks similar to the advertised route* | Any text which guides the reader on how to access other walks or tells the reader to walk certain other routes such as ‘detailed Guides for these routes are available from Tourist Information Centres and from www.exe-estuary.org.’  Do not include in this category text which only states it is easy to continue doing similar walks (code 75). Also do not include in this category text which merely promotes further similar walks e.g. ‘other similar leaflets are available’ (code 52). |
| 77. Enhancing self-efficacy → The advertised walk → Reinforcement → Modelling  *Modelling walking on the advertised route pictorially* | Any picture which depicts people walking part of the advertised route.  Do not include in this category pictures of walking/exercising where it is not clear whether it is related to the advertised walk (code 68). |
| 78. Enhancing self-efficacy → The advertised walk → Route difficulties → Encouraging terrain management  *Encouraging ways to overcome difficulties with the terrain on the advertised route* | Any text which conveys the **ease** with which one can overcome difficulties with the terrain on the advertised walk **without** guiding the reader on how to overcome them such as ‘the hills should not be a problem.’  Do not include in this category text which guides the reader on how to overcome difficulties with the terrain such as ‘walk well away from the edge of the cliff as they are prone to landslides’ (code 79). |
| 79. Enhancing self-efficacy → The advertised walk → Route difficulties → Guidance for terrain management  *Guidance on ways to overcome difficulties with the terrain on the advertised route* | Any text which guides the reader on how to overcome difficulties with the terrain on the advertised walk such as ‘this is a tidal crossing which cannot be passed within 2 hours either side of high tide, and you should consult the tide times website (see the back page of this leaﬂet) before crossing onto the Bere Peninsula.’  Do not include in this category text which only states that it is easy to overcome difficulties with the terrain (code 78). Also do not include in this category text which merely promotes overcoming terrain difficulties such as ‘take care as there are no pavements’ (code 53). |
| 80. Enhancing self-efficacy → The advertised walk → Route difficulties → Encouraging appropriate equipment  *Encouraging equipment necessary for the advertised route* | Any text which conveys how appropriate equipment is easy to access for the purposes of the advertised walk ‘getting some stout footwear should be easy enough.’  Do not include in this category text which guides the reader on appropriate equipment to take such as ‘you must take water with you even on a cloudy day’ or ‘a robust pair of walking boots should be taken in order to manage the hills’ (code 81). |
| 81. Enhancing self-efficacy → The advertised walk → Route difficulties → Guidance for appropriate equipment  *Guidance on equipment necessary for the advertised route* | Any text which guides the reader on how to overcome difficulties on the advertised walk by using appropriate equipment or clothing such as ‘you must wear good quality walking boots to reduce the risk of falling.’  Do not include in this category text which only states how appropriate equipment will make the walk easier such as ‘wearing more layers will make this walk easier on a wintery day’ (code 80). Also do not include in this category text which merely promotes appropriate equipment such as ‘consider wearing good shoes’ (code 54). |
| 82. Enhancing self-efficacy → The advertised walk → Route difficulties → Encouraging map reading  *Encouraging map reading* | Any text which conveys how map reading is **easy** **without** guiding the reader to read the map such as ‘following the direction on the map should not be difficult.’  Do not include in this category, text that guides the reader on how to read the maps in the leaflet such as ‘follow the route marked red to make sure you stay on track’ (code 83). |
| 83. Enhancing self-efficacy → The advertised walk → Route difficulties → Guidance for map reading  *Guidance on map reading* | Any text which guides the reader to use or read the map in a certain way such as ‘check the map every now and again to make sure you are on the right path’ or more imperative statements such as ‘follow the red marked route on the map to make sure you stay on track.’  Do not include in this category text which only states that reading the maps will make the walk easier such as ‘following the map directions is easy’ (code 82). Also do not include in this category text which merely promotes map reading such as ‘use the maps within’ (code 55). |
| 84. Enhancing self-efficacy → The advertised walk → Route difficulties → Encouraging direction taking  *Encouraging direction taking for the advertised route* | Any text which conveys to the reader that following the directions in the leaflet/booklet is easy such as ‘this booklet makes the route easy to follow.’  Do not include in this category text which guides the reader on directions to take such as ‘after passing the old wall of St. Katherine’s Priory on the right, cross Prince Charles Road and walk up St. Katherine’s Road’ (code 85). |
| 85. Enhancing self-efficacy → The advertised walk → Route difficulties → Guidance for direction taking  *Guidance for direction taking on the advertised route* | Any text which either guides the reader to follow directions or outlines or guides specific directions to take. In the case of the former, text could include statements such as ‘make sure you follow each step carefully to ensure you follow the route correctly’ or ‘read each direction before you set off.’ In the case of the latter, text can include guidance such as ‘start at junction of Pennsylvania Rd’ or ‘turn left at end of public footpath and go straight ahead along Velwell Rd.’ These sorts of direction taking guidance are frequent in walking leaflets and booklets.  Do not include in this category text which only states that following directions is easy such as ‘with the help of this leaflet, it should be easy to follow the route’ (code 84). Also do not include in this category text which merely promotes following directions such as ‘you can consider the directions which are described in this booklet’ (code 56). |
| 86. Enhancing self-efficacy → The advertised walk → Route difficulties → Encouraging barrier reduction  *Encouraging ways to reduce barriers to walking the advertised route* | Any text that says that general barriers for completing the advertised walk are easy to overcome, without telling the reader **how** to overcome them. These barriers would **not** include references to terrain, appropriate equipment, map reading or direction taking. Text could include statements such as ‘it is easy to do the walk even in bad weather’ or ‘with a little bit of arranging public transport it is easy to get to the start of the walk.’ Both statements target barrier reduction (weather, access) without guiding on how to overcome the barriers.  Do not include in this category any text which guides the reader on how to overcome general barriers such as ‘buses on A376 Exmouth Road or trains from Lympstone can be used to shorten the walk if required’ (code 87). |
| 87. Enhancing self-efficacy → The advertised walk → Route difficulties → Guidance for barrier reduction  *Guidance on ways to reduce barriers to walking the advertised route* | Any text that guides the reader on how to overcome general barriers to completing the advertised walk. Text could include statements such as ‘take extra care to walk on drier sections of the path when the weather is bad’ or ‘free guided tours are offered.’ These provide guidance on barriers not mentioned elsewhere in the coding scheme (weather/cost).  Do not include in this category any text which merely says that adapting to barriers for the advertised walk is easy to do (code 86). Also do not include in this category text which merely promotes the reduction of general barriers to doing the advertised walk such as ‘the walk is suitable for wheelchair users and pushchairs’ (code 57) as this only addresses the existence of barriers for the disabled/those with pushchairs; it does not build confidence for doing the route in a wheelchair/with a pushchair. |
|  |  |
| 0. Uncoded Text  *Uncoded text* | Any textual statements not captured in the above coding scheme. This can include (but is not limited to) publishing credits, feedback forms, details about the locations that are not captured in the coding scheme (e.g. this beach was used in a film), other website information (if it is not used to promote maintenance), and information on environmental behaviours (e.g. take litter home, buy locally produced goods etc). |
