# Supplementary material for: How do brochures encourage walking in natural environments in the UK? A content analysis
Source: Health Promot Int. 2016 Oct 28;33(2):299–310. doi: 10.1093/heapro/daw083 (PMC5892139; doi:10.1093/heapro/daw083)
Supplement: Supplementary File S-4 [file daw083_supplementary_file_s-4.docx]

| **Category Number** | **Category Name** | ***n* (rater 1)** | ***n* (rater 2)** | ***n* (agreed)** | **AC1** | ***p*** | **LB** | **UB** |
| --- | --- | --- | --- | --- | --- | --- | --- | --- |
| 1 | Information about recommended physical activity guidelines | 0 | 0 | 0 | n/a | n/a | n/a | n/a |
| 2 | Information about the distance of the advertised route | 18 | 20 | 17 | 0.89 | <.001 | 0.47 | 1.00 |
| 3 | Information about the time it may take to complete the advertised route | 0 | 1 | 0 | -0.01 | 1.000 | -0.01 | -0.01 |
| 4 | Information about the terrain of the advertised route | 47 | 47 | 42 | 0.89 | <.001 | 0.63 | 1.00 |
| 5 | Presence of a map | 11 | 11 | 11 | 1.00 | <.001 | 0.41 | 1.00 |
| 6 | Information about the overall course of the advertised route | 38 | 13 | 8 | 0.31 | 0.003 | 0.09 | 0.52 |
| 7 | Information about maps related to the advertised route | 3 | 4 | 3 | 0.86 | 0.043 | -0.12 | 1.00 |
| 8 | Information about public transport options related to the advertised route | 20 | 21 | 15 | 0.73 | <.001 | 0.36 | 1.00 |
| 9 | Information about parking provision related to the advertised route | 3 | 3 | 3 | 1.00 | 0.043 | -0.14 | 1.00 |
| 10 | Information about the provision of toilets on the advertised route | 3 | 3 | 3 | 1.00 | 0.043 | -0.14 | 1.00 |
| 11 | Information about refreshments on, or at the end of, the advertised route | 6 | 7 | 6 | 0.92 | 0.007 | 0.18 | 1.00 |
| 12 | Financial consequences of walking | 2 | 2 | 2 | 1.00 | 0.081 | -0.32 | 1.00 |
| 13 | Environmental consequences of walking | 3 | 2 | 2 | 0.80 | 0.081 | -0.32 | 1.00 |
| 14 | Physical health consequences of walking | 3 | 3 | 3 | 1.00 | 0.043 | -0.14 | 1.00 |
| 15 | Mental health consequences of walking | 4 | 4 | 4 | 1.00 | 0.024 | 0.01 | 1.00 |
| 16 | Social benefits of walking | 1 | 1 | 1 | 1.00 | 0.161 | -0.98 | 1.00 |
| 17 | Benefits to children of walking | 0 | 0 | 0 | n/a | n/a | n/a | n/a |
| 18 | Viewing a monument as a consequence of walking the advertised route | 0 | 0 | 0 | n/a | n/a | n/a | n/a |
| 19 | Viewing historical points of interest as consequences of walking the advertised route | 57 | 42 | 40 | 0.81 | <.001 | 0.56 | 1.00 |
| 20 | Viewing wildlife as a consequence of walking the advertised route | 5 | 5 | 5 | 1.00 | 0.013 | 0.12 | 1.00 |
| 21 | Viewing scenery as a consequence of walking the advertised route | 13 | 23 | 11 | 0.61 | <.001 | 0.25 | 0.97 |
| 22 | Botanical points of interest as consequences of walking the advertised route | 2 | 2 | 2 | 1.00 | 0.081 | -0.40 | 1.00 |
| 23 | Social consequences of walking the advertised route | 0 | 0 | 0 | n/a | n/a | n/a | n/a |
| 24 | Benefits to children of walking the advertised route | 0 | 0 | 0 | n/a | n/a | n/a | n/a |
| 25 | Accommodation at the destination as a consequence of walking the advertised route | 3 | 4 | 3 | 0.86 | 0.043 | -0.12 | 1.00 |
| 26 | Leisure opportunities as consequences of walking the advertised route | 16 | 9 | 8 | 0.64 | 0.003 | 0.19 | 1.00 |
| 27 | Normative information about recommended physical activity guidelines or walking | 0 | 0 | 0 | n/a | n/a | n/a | n/a |
| 28 | Expert recommendations about physical activity or walking | 0 | 0 | 0 | n/a | n/a | n/a | n/a |
| 29 | Normative information about the financial consequences of walking | 0 | 0 | 0 | n/a | n/a | n/a | n/a |
| 30 | Normative information about the environmental consequences of walking | 1 | 1 | 1 | 1.00 | 0.161 | -0.98 | 1.00 |
| 31 | Normative information about the physical health consequences of walking | 0 | 0 | 0 | n/a | n/a | n/a | n/a |
| 32 | Normative information about the mental health consequences of walking | 0 | 0 | 0 | n/a | n/a | n/a | n/a |
| 33 | Normative information about the social consequences of walking | 0 | 0 | 0 | n/a | n/a | n/a | n/a |
| 34 | Normative information about the benefits to children of walking | 1 | 1 | 1 | 1.00 | 0.161 | -0.98 | 1.00 |
| 35 | Normative information about viewing a monument on the advertised route | 0 | 0 | 0 | n/a | n/a | n/a | n/a |
| 36 | Normative information about viewing historical points of interest on the advertised route | 0 | 0 | 0 | n/a | n/a | n/a | n/a |
| 37 | Normative information about viewing wildlife on the advertised route | 0 | 0 | 0 | n/a | n/a | n/a | n/a |
| 38 | Normative information about viewing scenery on the advertised route | 0 | 0 | 0 | n/a | n/a | n/a | n/a |
| 39 | Normative information about viewing botanical points of interest on the advertised route | 0 | 0 | 0 | n/a | n/a | n/a | n/a |
| 40 | Normative information about the social consequences of walking the advertised route | 0 | 0 | 0 | n/a | n/a | n/a | n/a |
| 41 | Normative information about the benefits to children of walking the advertised route | 0 | 0 | 0 | n/a | n/a | n/a | n/a |
| 42 | Normative information about accommodation at the destination of the advertised route | 0 | 0 | 0 | n/a | n/a | n/a | n/a |
| 43 | Normative information about leisure opportunities on, or at the end of, the advertised route | 0 | 0 | 0 | n/a | n/a | n/a | n/a |
| 44 | Prompting walking goals based on distance | 0 | 0 | 0 | n/a | n/a | n/a | n/a |
| 45 | Prompting walking goals based on time | 0 | 0 | 0 | n/a | n/a | n/a | n/a |
| 46 | Prompting barrier reduction for walking | 2 | 1 | 1 | 0.66 | 0.162 | -0.66 | 1.00 |
| 47 | Prompting repeated walking | 0 | 1 | 0 | -0.01 | 1.000 | -0.01 | -0.01 |
| 48 | Prompting distance goals for the advertised route | 0 | 0 | 0 | n/a | n/a | n/a | n/a |
| 49 | Prompting time goals for the advertised route | 1 | 0 | 0 | -0.01 | 1.000 | -0.01 | -0.01 |
| 50 | Presence of a map key | 3 | 2 | 2 | 0.80 | 0.081 | -0.32 | 1.00 |
| 51 | Prompting attention to signage on the advertised route | 0 | 0 | 0 | n/a | n/a | n/a | n/a |
| 52 | Prompting repeated recreational walking similar to the advertised route | 5 | 6 | 2 | 0.36 | 0.084 | -0.15 | 0.87 |
| 53 | Prompting ways to overcome difficulties with the terrain on the advertised route | 4 | 0 | 0 | -0.01 | 1.000 | -0.01 | -0.01 |
| 54 | Prompting equipment needed for walking the advertised route | 2 | 1 | 1 | 0.66 | 0.162 | -0.66 | 1.00 |
| 55 | Prompting map reading for the advertised route | 0 | 1 | 0 | -0.01 | 1.000 | -0.01 | -0.01 |
| 56 | Prompting direction taking for the advertised route | 2 | 5 | 2 | 0.57 | 0.082 | -0.02 | 1.00 |
| 57 | Prompting barrier reduction on the advertised route | 4 | 1 | 1 | 0.39 | 0.165 | -0.40 | 1.00 |
| 58 | Encouraging recommended levels of physical activity | 0 | 1 | 0 | -0.01 | 1.000 | -0.01 | -0.01 |
| 59 | Guidance on how to achieve recommended levels of physical activity | 1 | 1 | 0 | -0.01 | 1.000 | -0.01 | -0.01 |
| 60 | Encouraging walking goals based on distances | 0 | 1 | 0 | -0.01 | 1.000 | -0.01 | -0.01 |
| 61 | Guidance on walking goals based on distances | 1 | 0 | 0 | -0.01 | 1.000 | -0.01 | -0.01 |
| 62 | Encouraging walking goals based on time | 0 | 0 | 0 | n/a | n/a | n/a | n/a |
| 63 | Guidance on walking goals based on time | 1 | 0 | 0 | -0.01 | 1.000 | -0.01 | -0.01 |
| 64 | Encouraging the reduction of barriers to walking | 0 | 2 | 0 | -0.01 | 1.000 | -0.01 | -0.01 |
| 65 | Guidance on the reduction of barriers to walking | 3 | 2 | 2 | 0.80 | 0.081 | -0.32 | 1.00 |
| 66 | Encouraging repeated walking | 0 | 0 | 0 | n/a | n/a | n/a | n/a |
| 67 | Guidance on ways to continue walking | 0 | 0 | 0 | n/a | n/a | n/a | n/a |
| 68 | Modelling walking pictorially | 6 | 3 | 3 | 0.66 | 0.044 | -0.10 | 1.00 |
| 69 | Encouraging distance walking goals for the advertised route | 0 | 0 | 0 | n/a | n/a | n/a | n/a |
| 70 | Guidance on distance walking goals for the advertised route | 0 | 0 | 0 | n/a | n/a | n/a | n/a |
| 71 | Encouraging timed walking goals for the advertised route | 0 | 0 | 0 | n/a | n/a | n/a | n/a |
| 72 | Guidance on timed walking goals for the advertised route | 0 | 0 | 0 | n/a | n/a | n/a | n/a |
| 73 | Encouraging attention to signage on the advertised route | 1 | 2 | 0 | -0.01 | 1.000 | -0.01 | 0.01 |
| 74 | Guidance on attending to signage on the advertised route | 1 | 0 | 0 | -0.01 | 1.000 | -0.01 | -0.01 |
| 75 | Encouraging repeated recreational walks similar to the advertised route | 0 | 0 | 0 | n/a | n/a | n/a | n/a |
| 76 | Guidance on repeated recreational walks similar to the advertised route | 13 | 8 | 7 | 0.66 | 0.004 | 0.17 | 1.00 |
| 77 | Modelling walking on the advertised route pictorially | 0 | 0 | 0 | n/a | n/a | n/a | n/a |
| 78 | Encouraging ways to overcome difficulties with the terrain on the advertised route | 1 | 1 | 0 | -0.01 | 1.000 | -0.01 | -0.01 |
| 79 | Guidance on ways to overcome difficulties with the terrain on the advertised route | 4 | 5 | 1 | 0.21 | 0.170 | -0.23 | 0.65 |
| 80 | Encouraging equipment necessary for the advertised route | 0 | 0 | 0 | n/a | n/a | n/a | n/a |
| 81 | Guidance on equipment necessary for the advertised route | 0 | 0 | 0 | n/a | n/a | n/a | n/a |
| 82 | Encouraging map reading | 0 | 0 | 0 | n/a | n/a | n/a | n/a |
| 83 | Guidance on map reading | 1 | 1 | 1 | 1.00 | 0.161 | -0.98 | 1.00 |
| 84 | Encouraging direction taking for the advertised route | 1 | 1 | 0 | -0.01 | 1.000 | -0.01 | -0.01 |
| 85 | Guidance for direction taking on the advertised route | 148 | 153 | 143 | 0.95 | <.001 | 0.82 | 1.00 |
| 86 | Encouraging ways to reduce barriers to walking the advertised route | 0 | 0 | 0 | n/a | n/a | n/a | n/a |
| 87 | Guidance on ways to reduce barriers to walking the advertised route | 3 | 1 | 0 | -0.01 | 1.000 | -0.01 | -0.01 |
| 88 | Uncoded or missed text | 8 | 47 | 6 | 0.21 | 0.009 | 0.03 | 0.39 |
| Overall agreement at superordinate level = 80.67% | | 476 | 476 | 384 | 0.77 | <.001 | 0.73 | 0.82 |
| Overall agreement at general physical activity / advertised route level = 80.67% | | 476 | 476 | 384 | 0.79 | <.001 | 0.75 | 0.83 |
| Overall agreement at thematic grouping level =78.78% | | 476 | 476 | 375 | 0.78 | <.001 | 0.74 | 0.82 |
| Overall agreement at individual category level = 76.26% | | 476 | 476 | 363 | 0.76 | <.001 | 0.72 | 0.80 |

| **Category Number** | **Category Name** | ***n* (rater 1)** | ***n* (rater 2)** | ***n* (agreed)** | **AC1** | ***p*** | **LB** | **UB** |
| --- | --- | --- | --- | --- | --- | --- | --- | --- |
| 1 | Information about recommended physical activity guidelines | 0 | 0 | 0 | n/a | n/a | n/a | n/a |
| 2 | Information about the distance of the advertised route | 18 | 18 | 18 | 1.00 | <.001 | 0.54 | 1.00 |
| 3 | Information about the time it may take to complete the advertised route | 0 | 1 | 0 | -0.01 | 1.000 | -0.01 | -0.01 |
| 4 | Information about the terrain of the advertised route | 46 | 46 | 45 | 0.98 | <.001 | 0.70 | 1.00 |
| 5 | Presence of a map | 11 | 11 | 11 | 1.00 | <.001 | 0.41 | 1.00 |
| 6 | Information about the overall course of the advertised route | 26 | 26 | 26 | 1.00 | <.001 | 0.62 | 1.00 |
| 7 | Information about maps related to the advertised route | 3 | 3 | 3 | 1.00 | 0.043 | -0.14 | 1.00 |
| 8 | Information about public transport options related to the advertised route | 20 | 22 | 18 | 0.86 | <.001 | 0.46 | 1.00 |
| 9 | Information about parking provision related to the advertised route | 3 | 3 | 3 | 1.00 | 0.043 | -0.14 | 1.00 |
| 10 | Information about the provision of toilets on the advertised route | 3 | 3 | 3 | 1.00 | 0.043 | -0.14 | 1.00 |
| 11 | Information about refreshments on, or at the end of, the advertised route | 6 | 7 | 6 | 0.92 | 0.007 | 0.18 | 1.00 |
| 12 | Financial consequences of walking | 2 | 2 | 2 | 1.00 | 0.081 | -0.40 | 1.00 |
| 13 | Environmental consequences of walking | 3 | 3 | 3 | 1.00 | 0.043 | -0.14 | 1.00 |
| 14 | Physical health consequences of walking | 3 | 3 | 3 | 1.00 | 0.043 | -0.14 | 1.00 |
| 15 | Mental health consequences of walking | 4 | 4 | 4 | 1.00 | 0.024 | 0.01 | 1.00 |
| 16 | Social benefits of walking | 1 | 1 | 1 | 1.00 | 0.161 | -0.98 | 1.00 |
| 17 | Benefits to children of walking | 0 | 0 | 0 | n/a | n/a | n/a | n/a |
| 18 | Viewing a monument as a consequence of walking the advertised route | 0 | 0 | 0 | n/a | n/a | n/a | n/a |
| 19 | Viewing historical points of interest as consequences of walking the advertised route | 54 | 51 | 50 | 0.95 | <.001 | 0.70 | 1.00 |
| 20 | Viewing wildlife as a consequence of walking the advertised route | 5 | 5 | 5 | 1.00 | 0.013 | 0.12 | 1.00 |
| 21 | Viewing scenery as a consequence of walking the advertised route | 16 | 18 | 15 | 0.88 | <.001 | 0.44 | 1.00 |
| 22 | Botanical points of interest as consequences of walking the advertised route | 2 | 2 | 2 | 1.00 | 0.081 | -0.40 | 1.00 |
| 23 | Social consequences of walking the advertised route | 0 | 0 | 0 | n/a | n/a | n/a | n/a |
| 24 | Benefits to children of walking the advertised route | 0 | 0 | 0 | n/a | n/a | n/a | n/a |
| 25 | Accommodation at the destination as a consequence of walking the advertised route | 4 | 4 | 4 | 1.00 | 0.024 | 0.01 | 1.00 |
| 26 | Leisure opportunities as consequences of walking the advertised route | 16 | 10 | 9 | 0.69 | 0.001 | 0.24 | 1.00 |
| 27 | Normative information about recommended physical activity guidelines or walking | 0 | 0 | 0 | n/a | n/a | n/a | n/a |
| 28 | Expert recommendations about physical activity or walking | 0 | 0 | 0 | n/a | n/a | n/a | n/a |
| 29 | Normative information about the financial consequences of walking | 0 | 0 | 0 | n/a | n/a | n/a | n/a |
| 30 | Normative information about the environmental consequences of walking | 1 | 1 | 1 | 1.00 | 0.161 | -0.98 | 1.00 |
| 31 | Normative information about the physical health consequences of walking | 0 | 0 | 0 | n/a | n/a | n/a | n/a |
| 32 | Normative information about the mental health consequences of walking | 0 | 0 | 0 | n/a | n/a | n/a | n/a |
| 33 | Normative information about the social consequences of walking | 0 | 0 | 0 | n/a | n/a | n/a | n/a |
| 34 | Normative information about the benefits to children of walking | 1 | 1 | 1 | 1.00 | 0.161 | -0.98 | 1.00 |
| 35 | Normative information about viewing a monument on the advertised route | 0 | 0 | 0 | n/a | n/a | n/a | n/a |
| 36 | Normative information about viewing historical points of interest on the advertised route | 0 | 0 | 0 | n/a | n/a | n/a | n/a |
| 37 | Normative information about viewing wildlife on the advertised route | 0 | 0 | 0 | n/a | n/a | n/a | n/a |
| 38 | Normative information about viewing scenery on the advertised route | 0 | 0 | 0 | n/a | n/a | n/a | n/a |
| 39 | Normative information about viewing botanical points of interest on the advertised route | 0 | 0 | 0 | n/a | n/a | n/a | n/a |
| 40 | Normative information about the social consequences of walking the advertised route | 0 | 0 | 0 | n/a | n/a | n/a | n/a |
| 41 | Normative information about the benefits to children of walking the advertised route | 0 | 0 | 0 | n/a | n/a | n/a | n/a |
| 42 | Normative information about accommodation at the destination of the advertised route | 0 | 0 | 0 | n/a | n/a | n/a | n/a |
| 43 | Normative information about leisure opportunities on, or at the end of, the advertised route | 0 | 0 | 0 | n/a | n/a | n/a | n/a |
| 44 | Prompting walking goals based on distance | 0 | 0 | 0 | n/a | n/a | n/a | n/a |
| 45 | Prompting walking goals based on time | 0 | 0 | 0 | n/a | n/a | n/a | n/a |
| 46 | Prompting barrier reduction for walking | 2 | 1 | 1 | 0.66 | 0.162 | -0.66 | 1.00 |
| 47 | Prompting repeated walking | 0 | 1 | 0 | -0.01 | 1.000 | -0.01 | -0.01 |
| 48 | Prompting distance goals for the advertised route | 1 | 1 | 1 | 1.00 | 0.161 | -0.98 | 1.00 |
| 49 | Prompting time goals for the advertised route | 1 | 0 | 0 | -0.01 | 1.000 | -0.01 | -0.01 |
| 50 | Presence of a map key | 3 | 3 | 3 | 1.00 | 0.043 | -0.14 | 1.00 |
| 51 | Prompting attention to signage on the advertised route | 3 | 3 | 3 | 1.00 | 0.043 | -0.14 | 1.00 |
| 52 | Prompting repeated recreational walking similar to the advertised route | 4 | 4 | 4 | 1.00 | 0.024 | 0.01 | 1.00 |
| 53 | Prompting ways to overcome difficulties with the terrain on the advertised route | 4 | 1 | 1 | 0.39 | 0.165 | -0.40 | 1.00 |
| 54 | Prompting equipment needed for walking the advertised route | 2 | 2 | 2 | 1.00 | 0.081 | -0.40 | 1.00 |
| 55 | Prompting map reading for the advertised route | 0 | 1 | 0 | -0.01 | 1.000 | -0.01 | -0.01 |
| 56 | Prompting direction taking for the advertised route | 2 | 2 | 2 | 1.00 | 0.081 | -0.40 | 1.00 |
| 57 | Prompting barrier reduction on the advertised route | 3 | 3 | 3 | 1.00 | 0.043 | -0.14 | 1.00 |
| 58 | Encouraging recommended levels of physical activity | 0 | 1 | 0 | -0.01 | 1.000 | -0.01 | -0.01 |
| 59 | Guidance on how to achieve recommended levels of physical activity | 1 | 1 | 0 | -0.01 | 1.000 | -0.01 | -0.01 |
| 60 | Encouraging walking goals based on distances | 0 | 1 | 0 | -0.01 | 1.000 | -0.01 | -0.01 |
| 61 | Guidance on walking goals based on distances | 1 | 0 | 0 | -0.01 | 1.000 | -0.01 | -0.01 |
| 62 | Encouraging walking goals based on time | 0 | 0 | 0 | n/a | n/a | n/a | n/a |
| 63 | Guidance on walking goals based on time | 1 | 0 | 0 | -0.01 | 1.000 | -0.01 | -0.01 |
| 64 | Encouraging the reduction of barriers to walking | 0 | 1 | 0 | -0.01 | 1.000 | -0.01 | -0.01 |
| 65 | Guidance on the reduction of barriers to walking | 3 | 2 | 2 | 0.80 | 0.081 | -0.32 | 1.00 |
| 66 | Encouraging repeated walking | 0 | 0 | 0 | n/a | n/a | n/a | n/a |
| 67 | Guidance on ways to continue walking | 0 | 0 | 0 | n/a | n/a | n/a | n/a |
| 68 | Modelling walking pictorially | 6 | 6 | 6 | 1.00 | 0.007 | 0.20 | 1.00 |
| 69 | Encouraging distance walking goals for the advertised route | 0 | 0 | 0 | n/a | n/a | n/a | n/a |
| 70 | Guidance on distance walking goals for the advertised route | 0 | 0 | 0 | n/a | n/a | n/a | n/a |
| 71 | Encouraging timed walking goals for the advertised route | 0 | 0 | 0 | n/a | n/a | n/a | n/a |
| 72 | Guidance on timed walking goals for the advertised route | 0 | 0 | 0 | n/a | n/a | n/a | n/a |
| 73 | Encouraging attention to signage on the advertised route | 2 | 2 | 1 | 0.49 | 0.164 | -0.49 | 1.00 |
| 74 | Guidance on attending to signage on the advertised route | 1 | 1 | 1 | 1.00 | 0.161 | -0.98 | 1.00 |
| 75 | Encouraging repeated recreational walks similar to the advertised route | 0 | 0 | 0 | n/a | n/a | n/a | n/a |
| 76 | Guidance on repeated recreational walks similar to the advertised route | 15 | 15 | 14 | 0.93 | <.001 | 0.45 | 1.00 |
| 77 | Modelling walking on the advertised route pictorially | 0 | 0 | 0 | n/a | n/a | n/a | n/a |
| 78 | Encouraging ways to overcome difficulties with the terrain on the advertised route | 2 | 3 | 2 | 0.80 | 0.081 | -0.32 | 1.00 |
| 79 | Guidance on ways to overcome difficulties with the terrain on the advertised route | 2 | 2 | 2 | 1.00 | 0.081 | -0.40 | 1.00 |
| 80 | Encouraging equipment necessary for the advertised route | 0 | 0 | 0 | n/a | n/a | n/a | n/a |
| 81 | Guidance on equipment necessary for the advertised route | 0 | 0 | 0 | n/a | n/a | n/a | n/a |
| 82 | Encouraging map reading | 0 | 0 | 0 | n/a | n/a | n/a | n/a |
| 83 | Guidance on map reading | 1 | 1 | 1 | 1.00 | 0.161 | -0.98 | 1.00 |
| 84 | Encouraging direction taking for the advertised route | 1 | 1 | 0 | -0.01 | 1.000 | -0.01 | -0.01 |
| 85 | Guidance for direction taking on the advertised route | 148 | 153 | 148 | 0.98 | <.001 | 0.85 | 1.00 |
| 86 | Encouraging ways to reduce barriers to walking the advertised route | 0 | 0 | 0 | n/a | n/a | n/a | n/a |
| 87 | Guidance on ways to reduce barriers to walking the advertised route | 1 | 1 | 0 | -0.01 | 1.000 | -0.01 | -0.01 |
| 88 | Uncoded or missed text | 18 | 18 | 18 | 1.00 | <.001 | 0.54 | 1.00 |
| Overall agreement at the superordinate level = 96.22% | | 476 | 476 | 458 | 0.96 | <.001 | 0.94 | 0.98 |
| Overall agreement at the general physical activity / advertised route level = 96.22% | | 476 | 476 | 458 | 0.96 | <.001 | 0.94 | 0.98 |
| Overall agreement at the thematic grouping level = 94.54% | | 476 | 476 | 450 | 0.94 | <.001 | 0.92 | 0.96 |
| Overall agreement at the individual category level = 94.12% | | 476 | 476 | 448 | 0.94 | <.001 | 0.92 | 0.96 |
