# Supplementary material for: How do brochures encourage walking in natural environments in the UK? A content analysis
Source: Health Promot Int. 2016 Oct 28;33(2):299–310. doi: 10.1093/heapro/daw083 (PMC5892139; doi:10.1093/heapro/daw083)
Supplement: Supplementary File S-5 [file daw083_supplementary_file_s-5.docx]

|  |  |  |  |  |  |  |
| --- | --- | --- | --- | --- | --- | --- |
|  | % brochures with ≥1 instance | *M* instances | *SD* instances | Max instances | % all content | % of superordinate |
| *Providing information* | 100.0 | 57.12 | 72.83 | 299 | 29.1 | - |
| 1. Information on recommended physical activity guidelines | 3.8 | 0.04 | 0.20 | 1 | 0.0 | 0.1 |
| 2. Information on the distance of the advertised route | 92.3 | 8.50 | 10.75 | 44 | 4.3 | 14.9 |
| 3. Information on the length it may take to complete the advertised route | 26.9 | 1.04 | 2.95 | 14 | 0.5 | 1.8 |
| 4. Information on the terrain of the advertised route | 84.6 | 8.77 | 14.30 | 48 | 4.5 | 15.4 |
| 5. Presence of a map | 84.6 | 4.08 | 3.91 | 12 | 2.1 | 7.1 |
| 6. Information on the overall course of the advertised route | 88.5 | 15.12 | 20.97 | 91 | 7.7 | 26.5 |
| 7. Information on maps related to the advertised route | 50.0 | 2.08 | 4.28 | 19 | 1.1 | 3.6 |
| 8. Information on public transport options related to the advertised route | 61.5 | 9.77 | 14.77 | 54 | 5.0 | 17.1 |
| 9. Information on parking provision related to the advertised route | 38.5 | 1.62 | 2.98 | 9 | 0.8 | 2.8 |
| 10. Information about provision of toilets on the advertised route | 46.2 | 1.81 | 4.04 | 18 | 0.9 | 3.2 |
| 11. Information on refreshments on, or at the end of, the advertised route | 57.7 | 4.31 | 7.77 | 29 | 2.2 | 7.5 |
| *Highlighting consequences* | 100.0 | 50.73 | 72.73 | 361 | 25.9 | - |
| 12. Financial consequences of walking | 7.7 | 0.12 | 0.43 | 2 | 0.1 | 0.2 |
| 13. Environmental consequences of walking | 7.7 | 0.15 | 0.61 | 3 | 0.1 | 0.3 |
| 14. Physical health consequences of walking | 7.7 | 0.23 | 0.82 | 3 | 0.1 | 0.5 |
| 15. Mental health consequences of walking | 7.7 | 0.27 | 0.96 | 4 | 0.1 | 0.5 |
| 16. Social benefits of walking | 3.8 | 0.04 | 0.20 | 1 | 0.0 | 0.1 |
| 17. Benefits to children of walking | 3.8 | 0.04 | 0.20 | 1 | 0.0 | 0.1 |
| 18. Viewing a monument as a consequence of walking the advertised route | 50.0 | 1.69 | 3.02 | 13 | 0.9 | 3.3 |
| 19. Viewing historical features as consequences of walking the advertised route | 100.0 | 25.50 | 38.51 | 187 | 13.0 | 50.3 |
| 20. Viewing wildlife as a consequence of walking the advertised route | 76.9 | 3.27 | 4.03 | 20 | 1.7 | 6.4 |
| 21. Viewing scenery as a consequence of walking the advertised route | 92.3 | 11.54 | 19.92 | 98 | 5.9 | 22.7 |
| 22. Botanical points of interest as consequences of walking the advertised route | 76.9 | 2.31 | 2.94 | 13 | 1.2 | 4.5 |
| 23. Social consequences of walking the advertised route | 3.8 | 0.04 | 0.20 | 1 | 0.0 | 0.1 |
| 24. Benefits to children of walking the advertised route | 11.5 | 0.12 | 0.33 | 1 | 0.1 | 0.2 |
| 25. Accommodation at the destination as a consequence of walking the advertised route | 23.1 | 0.65 | 1.55 | 6 | 0.3 | 1.3 |
| 26. Leisure opportunities as consequences of walking the advertised route | 42.3 | 4.77 | 8.42 | 30 | 2.4 | 9.4 |
| *Establishing normative beliefs* | 15.4 | 0.31 | 0.79 | 3 | 0.2 | - |
| 27. Normative information about recommended physical activity guidelines or walking | 3.8 | 0.04 | 0.20 | 1 | 0.0 | 12.5 |
| 28. Expert recommendations on physical activity or walking | 3.8 | 0.04 | 0.20 | 1 | 0.0 | 12.5 |
| 29. Normative information on the financial consequences of walking | 0.0 | 0.00 | 0.00 | 0 | 0.0 | 0.0 |
| 30. Normative information on the environmental consequences of walking | 3.8 | 0.04 | 0.20 | 1 | 0.0 | 12.5 |
| 31. Normative information on the physical health consequences of walking | 3.8 | 0.04 | 0.20 | 1 | 0.0 | 12.5 |
| 32. Normative information on the mental health consequences of walking | 0.0 | 0.00 | 0.00 | 0 | 0.0 | 0.0 |
| 33. Normative information on the social consequences of walking | 0.0 | 0.00 | 0.00 | 0 | 0.0 | 0.0 |
| 34. Normative information on the benefits to children of walking | 3.8 | 0.04 | 0.20 | 1 | 0.0 | 12.5 |
| 35. Normative information on viewing a monument on the advertised route | 0.0 | 0.00 | 0.00 | 0 | 0.0 | 0.0 |
| 36. Normative information on viewing historical points of interest on the advertised route | 3.8 | 0.04 | 0.20 | 1 | 0.0 | 12.5 |
| 37. Normative information on viewing wildlife on the advertised route | 0.0 | 0.00 | 0.00 | 0 | 0.0 | 0.0 |
| 38. Normative information on viewing scenery on the advertised route | 3.8 | 0.04 | 0.20 | 1 | 0.0 | 12.5 |
| 39. Normative information on viewing botanical points of interest on the advertised route | 0.0 | 0.00 | 0.00 | 0 | 0.0 | 0.0 |
| 40. Normative information on the social consequences of walking the advertised route | 0.0 | 0.00 | 0.00 | 0 | 0.0 | 0.0 |
| 41. Normative information on the benefits to children of walking the advertised route | 0.0 | 0.00 | 0.00 | 0 | 0.0 | 0.0 |
| 42. Normative information about accommodation at the destination of the advertised route | 0.0 | 0.00 | 0.00 | 0 | 0.0 | 0.0 |
| 43. Normative information about leisure opportunities on, or at the end of, the advertised route | 3.8 | 0.04 | 0.20 | 1 | 0.0 | 12.5 |
| *Promoting Intentions and Planning* | 100.0 | 10.73 | 13.11 | 55 | 5.5 | - |
| 44. Prompting walking goals based on distance | 7.7 | 0.19 | 0.69 | 3 | 0.1 | 1.8 |
| 45. Prompting walking goals based on time | 0.0 | 0.00 | 0.00 | 0 | 0.0 | 0.0 |
| 46. Prompting barrier reduction for walking | 7.7 | 0.12 | 0.43 | 2 | 0.1 | 1.1 |
| 47. Prompting repeated walking | 0.0 | 0.00 | 0.00 | 0 | 0.0 | 0.0 |
| 48. Prompting distance goals for the advertised route | 26.9 | 0.62 | 1.30 | 5 | 0.3 | 5.7 |
| 49. Prompting time goals for the advertised route | 23.1 | 0.50 | 1.27 | 6 | 0.3 | 4.7 |
| 50. Map key | 65.4 | 0.96 | 0.82 | 2 | 0.5 | 9.0 |
| 51. Prompting attention to signage on the advertised route | 38.5 | 1.04 | 1.84 | 8 | 0.5 | 9.7 |
| 52. Prompting repeated recreational walking similar to the advertised route | 73.1 | 4.04 | 5.23 | 17 | 2.1 | 37.6 |
| 53. Prompting ways to overcome difficulties with the terrain on the advertised route | 34.6 | 1.42 | 2.79 | 12 | 0.7 | 13.3 |
| 54. Prompting equipment needed for walking the advertised route | 11.5 | 0.12 | 0.33 | 1 | 0.1 | 1.1 |
| 55. Prompting map reading for the advertised route | 19.2 | 0.27 | 0.67 | 3 | 0.1 | 2.5 |
| 56. Prompting direction taking for the advertised route | 19.2 | 0.27 | 0.60 | 2 | 0.1 | 2.5 |
| 57. Prompting barrier reduction on the advertised route | 34.6 | 1.19 | 2.77 | 13 | 0.6 | 11.1 |
| *Enhancing self-efficacy* | 100.0 | 69.31 | 107.36 | 403 | 35.3 | - |
| 58. Encouraging recommended levels of physical activity | 0.0 | 0.00 | 0.00 | 0 | 0.0 | 0.0 |
| 59. Guidance on how to achieve recommended levels of physical activity | 3.8 | 0.04 | 0.20 | 1 | 0.0 | 0.1 |
| 60. Encouraging walking goals based on distances | 0.0 | 0.00 | 0.00 | 0 | 0.0 | 0.0 |
| 61. Guidance on walking goals based on distances | 7.7 | 0.08 | 0.27 | 1 | 0.0 | 0.1 |
| 62. Encouraging walking goals based on time | 0.0 | 0.00 | 0.00 | 0 | 0.0 | 0.0 |
| 63. Guidance on walking goals based on time | 3.8 | 0.04 | 0.20 | 1 | 0.0 | 0.1 |
| 64. Encouraging the reduction of barriers to walking | 0.0 | 0.00 | 0.00 | 0 | 0.0 | 0.0 |
| 65. Guidance on the reduction of barriers to walking | 3.8 | 0.12 | 0.59 | 3 | 0.1 | 0.2 |
| 66. Encouraging repeated walking | 0.0 | 0.00 | 0.00 | 0 | 0.0 | 0.0 |
| 67. Guidance on ways to continue walking | 0.0 | 0.00 | 0.00 | 0 | 0.0 | 0.0 |
| 68. Modelling physical activity pictorially | 3.8 | 0.04 | 0.20 | 1 | 0.0 | 0.1 |
| 69. Encouraging distance walking goals for the advertised route | 3.8 | 0.04 | 0.20 | 1 | 0.0 | 0.1 |
| 70. Guidance on distance walking goals for the advertised route | 26.9 | 0.42 | 0.86 | 3 | 0.2 | 0.6 |
| 71. Encouraging timed walking goals for the advertised route | 3.8 | 0.04 | 0.20 | 1 | 0.0 | 0.1 |
| 72. Guidance on timed walking goals for the advertised route | 7.7 | 0.92 | 4.51 | 23 | 0.5 | 1.3 |
| 73. Encouraging attention to signage on the advertised route | 3.8 | 0.08 | 0.39 | 2 | 0.0 | 0.1 |
| 74. Guidance on attending to signage on the advertised route | 19.2 | 0.19 | 0.40 | 1 | 0.1 | 0.3 |
| 75. Encouraging repeated recreational walks similar to the advertised route | 0.0 | 0.00 | 0.00 | 0 | 0.0 | 0.0 |
| 76. Guidance on repeated recreational walks similar to the advertised route | 46.2 | 2.50 | 4.58 | 20 | 1.3 | 3.6 |
| 77. Modelling walking on the advertised route pictorially | 30.8 | 1.35 | 2.86 | 12 | 0.7 | 1.9 |
| 78. Encouraging ways to overcome difficulties with the terrain on the advertised route | 3.8 | 0.04 | 0.20 | 1 | 0.0 | 0.1 |
| 79. Guidance on ways to overcome difficulties with the terrain on the advertised route | 53.8 | 1.92 | 2.45 | 8 | 1.0 | 2.8 |
| 80. Encouraging equipment necessary for the advertised route | 0.0 | 0.00 | 0.00 | 0 | 0.0 | 0.0 |
| 81. Guidance on equipment necessary for the advertised route | 15.4 | 0.23 | 0.65 | 3 | 0.1 | 0.3 |
| 82. Encouraging map reading | 0.0 | 0.00 | 0.00 | 0 | 0.0 | 0.0 |
| 83. Guidance on map reading | 7.7 | 0.12 | 0.43 | 2 | 0.1 | 0.2 |
| 84. Encouraging direction taking for the advertised route | 11.5 | 0.15 | 0.46 | 2 | 0.1 | 0.2 |
| 85. Guidance for direction taking on the advertised walk | 88.5 | 60.88 | 99.31 | 362 | 31.0 | 87.8 |
| 86. Encouraging ways to reduce barriers to walking the advertised route | 0.0 | 0.00 | 0.00 | 0 | 0.0 | 0.0 |
| 87. Guidance on ways to reduce barriers to walking the advertised route | 11.5 | 0.12 | 0.33 | 1 | 0.1 | 0.2 |
| *Text which was unable to be otherwise categorised* | 88.5 | 7.92 | 8.33 | 28 | 4.0 | - |
| “% leaflets with ≤1 instance” refers to the percentage of brochures in the sample with greater than or equal to one instance of the persuasive technique or superordinate.  “*M* instances” refers to the average number of instances of the persuasive technique or superordinate across the sample of brochures.  “*SD* instances” refers to the standard deviation of instances of the persuasive technique or superordinate across the sample of brochures.  “Max instances” refers to the maximum number of instances of the persuasive technique or superordinate in any one brochure in the sample. “% all content” refers to the percentage of all content which is accounted for by the persuasive technique or superordinate. “% of superordinate” refers to the percentage of superordinate content which is accounted for by the persuasive technique. | | | | | | |
